# Influence of weighted downhill running training on serial sarcomere number and work loop performance in the rat soleus

**DOI:** 10.1242/bio.059491

**Published:** 2022-07-25

**Authors:** Avery Hinks, Kaitlyn Jacob, Parastoo Mashouri, Kyle D. Medak, Martino V. Franchi, David C. Wright, Stephen H. M. Brown, Geoffrey A. Power

**Affiliations:** 1 Department of Human Health and Nutritional Sciences, College of Biological Sciences, University of Guelph, 50 Stone Road East, Guelph, ON N1G 2W1, Canada; 2 Department of Biomedical Sciences, Neuromuscular Physiology Laboratory, University of Padua, Padua 35122, Italy; 3 School of Kinesiology, Faculty of Land and Food Systems, University of British Columbia, Vancouver, BC V6T 1Z4, Canada

**Keywords:** Sarcomerogenesis, Eccentric training, Muscle architecture, Force-length relationship, Dynamic performance, Collagen

## Abstract

Increased serial sarcomere number (SSN) has been observed in rats following downhill running training due to the emphasis on active lengthening contractions; however, little is known about the influence on dynamic contractile function. Therefore, we employed 4 weeks of weighted downhill running training in rats, then assessed soleus SSN and work loop performance. We hypothesised trained rats would produce greater net work output during work loops due to a greater SSN. Thirty-one Sprague-Dawley rats were assigned to a training or sedentary control group. Weight was added during downhill running via a custom-made vest, progressing from 5–15% body mass. Following sacrifice, the soleus was dissected, and a force-length relationship was constructed. Work loops (cyclic muscle length changes) were then performed about optimal muscle length (L_O_) at 1.5–3-Hz cycle frequencies and 1–7-mm length changes. Muscles were then fixed in formalin at L_O_. Fascicle lengths and sarcomere lengths were measured to calculate SSN. Intramuscular collagen content and crosslinking were quantified via a hydroxyproline content and pepsin-solubility assay. Trained rats had longer fascicle lengths (+13%), greater SSN (+8%), and a less steep passive force-length curve than controls (*P*<0.05). There were no differences in collagen parameters (*P*>0.05). Net work output was greater (+78–209%) in trained than control rats for the 1.5-Hz work loops at 1 and 3-mm length changes (*P*<0.05), however, net work output was more related to maximum specific force (R^2^=0.17-0.48, *P*<0.05) than SSN (R^2^=0.03-0.07, *P*=0.17-0.86). Therefore, contrary to our hypothesis, training-induced sarcomerogenesis likely contributed little to the improvements in work loop performance.

This article has an associated First Person interview with the first author of the paper.

## Abstract (precis)

**Summary:** An investigation of adaptations in mechanical function induced by a novel method of weighted downhill running training in rats, and the connections to adaptations in muscle architecture.

## INTRODUCTION

Skeletal muscle remodels and adapts in response to specific conditions, as observed across the hierarchy of muscle structural organisation (Gans and Bock, 1965; Gans and de Vree, 1987; Jorgenson et al., 2020). An example is longitudinal skeletal muscle growth following downhill running training, seen as an increase in serial sarcomere number (SSN), a process termed sarcomerogenesis, due to the emphasis on active lengthening (eccentric) contractions (Butterfield et al., 2005; Chen et al., 2020; Lynn et al., 1998). During unaccustomed eccentric exercise, sarcomeres are overstretched, resulting in suboptimal actin-myosin overlap. The most supported hypothesis for sarcomerogenesis following eccentric training is that an increase in SSN occurs to re-establish optimal crossbridge overlap regions (Butterfield et al., 2005; Herring et al., 1984). While the influence of increased SSN on isometric contractile function is well-established (Lynn et al., 1998; Williams and Goldspink, 1978), less is known about the impact on dynamic contractile function.

A physiologically relevant assessment of dynamic performance *in vitro* is the ‘work loop’: sinusoidal cycles of muscle shortening and lengthening with phasic bursts of stimulation meant to simulate locomotion (Josephson and Darrell, 1989; Schaeffer and Lindstedt, 2013). Work loops can incorporate various cycle frequencies (i.e. speeds of shortening/lengthening) and muscle length changes, and are therefore influenced by muscle force-velocity and force-length properties (Josephson, 1999). Hence, there are optimal length changes and cycle frequencies for work loop performance, wherein active tension is as high as possible during shortening and as low as possible during subsequent lengthening (i.e. generates greater net work output) (Josephson and Darrell, 1989; Swoap et al., 1997). Sarcomerogenesis may place sarcomeres closer to optimal length throughout the range of motion, and allow individual sarcomeres to shorten slower, optimising force production across a wider range of muscle lengths based on the force-length and force-velocity relationships (Akagi et al., 2020; Drazan et al., 2019). SSN is also proportional to absolute maximum shortening velocity (Wickiewicz et al., 1984). Taken together, sarcomerogenesis may improve work loop performance, particularly in cycles with longer length changes and faster cycle frequencies.

Contrary to the above hypothesis, Cox et al. (2000) increased SSN of the rabbit latissimus dorsi via 3 weeks of incremental surgical stretch and observed a decrease in maximum work loop power output compared to controls. They attributed this impaired work loop performance to increased intramuscular collagen content caused by the surgical stretch. Increased collagen content and crosslinking enhance muscle passive tension during stretch (Brashear et al., 2020; Huijing, 1999; Kjær, 2004; Turrina et al., 2013), which can increase work in the lengthening phase of the work loop and thereby decrease net work output. While collagen content and mRNAs and growth factors related to collagen synthesis can increase in the rat medial gastrocnemius and quadriceps following isometric, concentric, and eccentric-based training (Han et al., 1999; Heinemeier et al., 2007; Zimmerman et al., 1993), collagen content and key enzymes related to collagen synthesis do not change in the rat soleus following short-term downhill running (Han et al., 1999) or long-term running training (Zimmerman et al., 1993). Collagen crosslinking was also shown to not change in the rat soleus with long-term running training (Zimmerman et al., 1993). Hence, the rat soleus in downhill running training may be a good model to assess the influence of sarcomerogenesis on work loop performance in the absence of increased collagen content.

Previous studies have employed downhill running in rodents with primarily an endurance training stimulus: on consecutive days with no progressive weighted overload (Butterfield et al., 2005; Chen et al., 2020). A stronger stimulus provided by progressively loaded training may have a more pronounced impact on muscle architecture and mechanical function (Butterfield and Herzog, 2006; Farup et al., 2012). Furthermore, running on many consecutive days may minimise time for recovery and hence remodelling between exercise bouts (Hyldahl and Hubal, 2014). This distinction in training stimuli was offered previously as explanation for observations of only small adaptations in rat soleus SSN and mechanical function following downhill running training (Chen et al., 2020). Larger magnitude changes in muscle architecture and strength in animals following 3 days/week training programs support this perspective as well (Butterfield and Herzog, 2006; Ochi et al., 2007; Zhu et al., 2021).

**Fig. 1.**
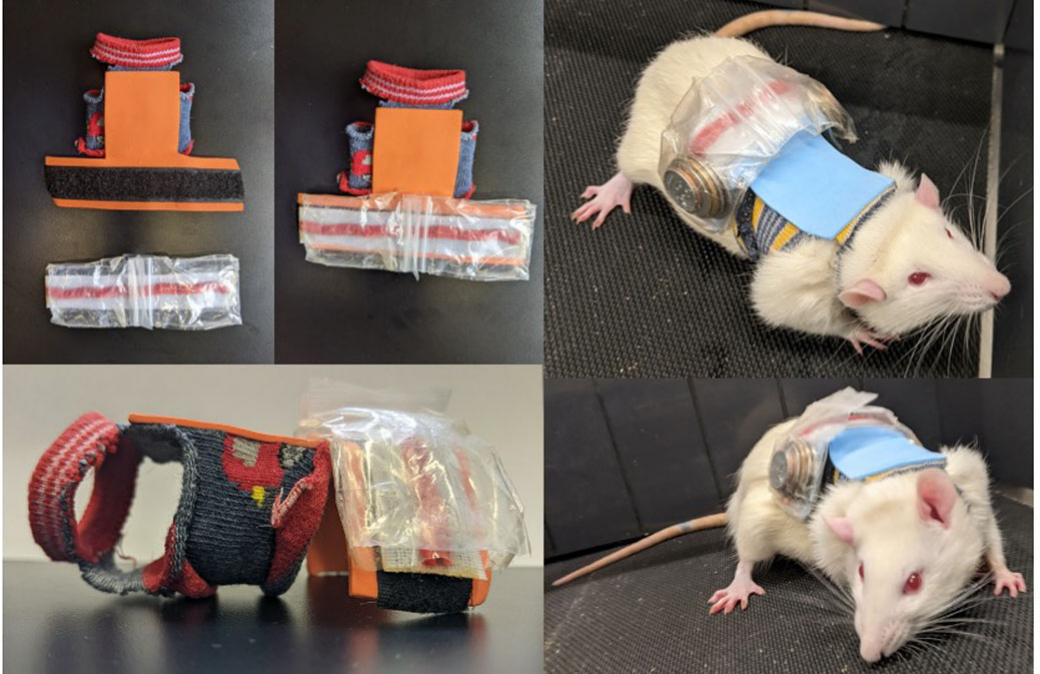
Rat weighted vest design (left) and a rat wearing a vest containing 15% of its body mass at week 4 of training (right).

To enhance the effect of downhill running training on muscle architecture, the present study employed 4 weeks of downhill running 3 days/week and progressively increased the eccentric stimulus during running via a novel model incorporating weighted vests (Fig. 1). The purposes were to: (1) assess how soleus SSN and intramuscular collagen adapt to eccentric training; and (2) investigate the influence on work loop performance. We hypothesised that soleus SSN would increase, and collagen content and crosslinking would not change following training. We also hypothesised that, due to training-induced sarcomerogenesis, work loop performance would improve, particularly in work loops with longer length changes and faster cycle frequencies.

## RESULTS

### Body weight, muscle weight, and physiological cross-sectional area (PCSA)

**Fig. 2.**
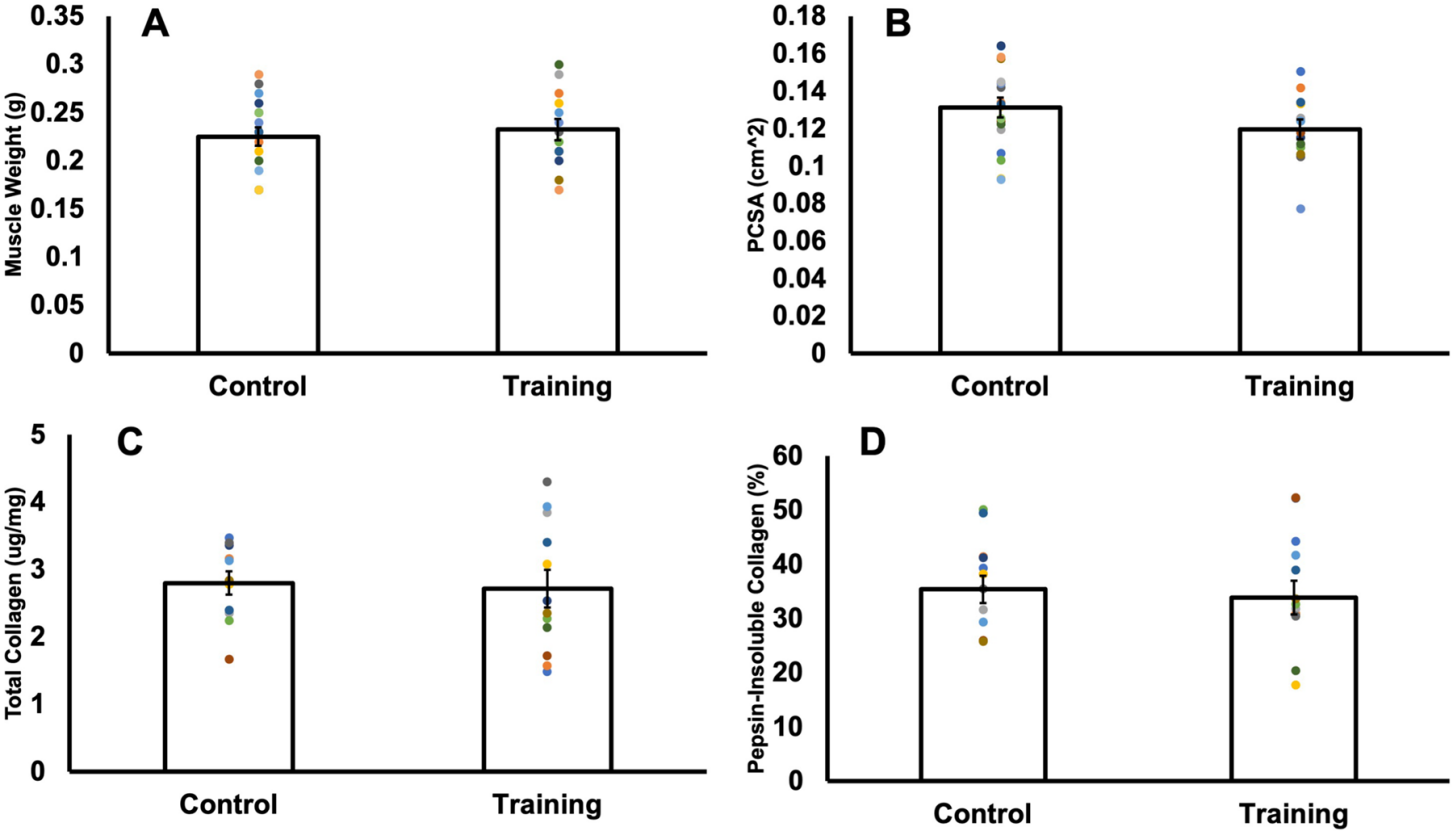
**Comparison of muscle wet weight (A), physiological cross-sectional area (PCSA) (B), total collagen concentration (C), and the percentage represented by pepsin-insoluble collagen (D) in control versus trained rats.** Data are reported as mean±s.e. (A,B: *n*=18 control, *n*=13 training; C,D: *n*=11 control, *n*=12 training). No significant differences (*P*>0.05) were found between control and training groups for any of these variables.

As shown in Fig. S1, there were no differences in body weight between control and trained rats at any time points. As shown in Fig. 2A and B, there were also no differences in muscle wet weight (*P*=0.62) or PCSA between trained and control rats (*P*=0.14).

### Collagen content and crosslinking

There were no differences in total hydroxyproline concentration (*P*=0.81) (Fig. 2C) or percent pepsin-insoluble collagen (*P*=0.71) (Fig. 2D) between trained and control rats, indicating no differences in collagen content or the relative amount of crosslinked collagen, respectively.

### Longitudinal muscle architecture

**Fig. 3.**
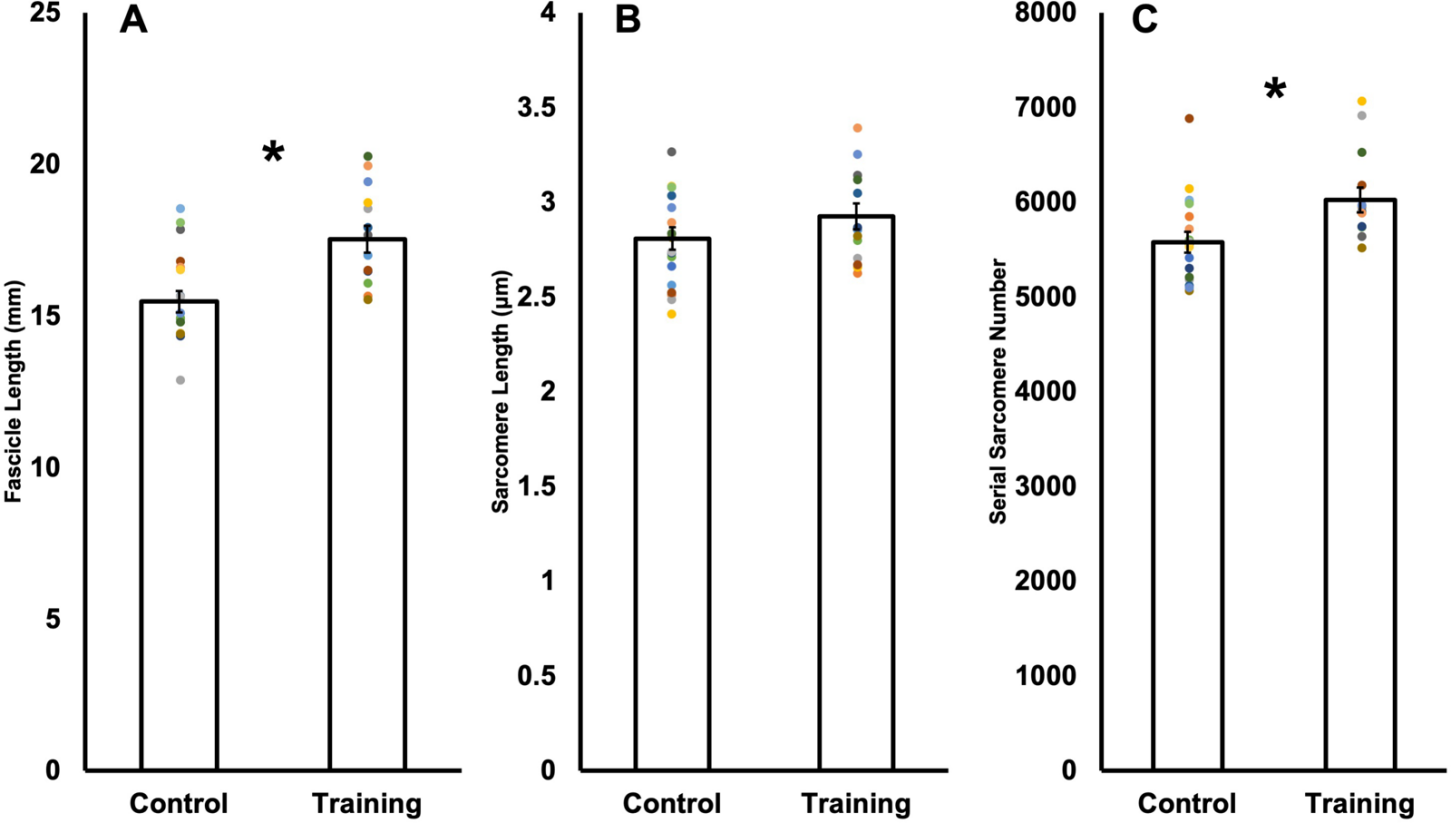
**Comparison of fascicle length (A), sarcomere length (B), and serial sarcomere number (C) in control versus trained rats.** Data are reported as mean±s.e. (*n*=18 control, *n*=13 training). *Significant difference (*P*<0.05) between control and training.

For average fascicle length (FL), 95% power was achieved with 18 control and 13 trained rats. FL was 13% longer (*P*<0.01, *d*=1.38) in trained [17.52±1.60 mm, 95% CI (16.55,18.49)] than control rats [15.47±1.49 mm, 95% CI (14.72,16.21)], implying a training-induced increase in FL (Fig. 3A).

**Fig. 4.**
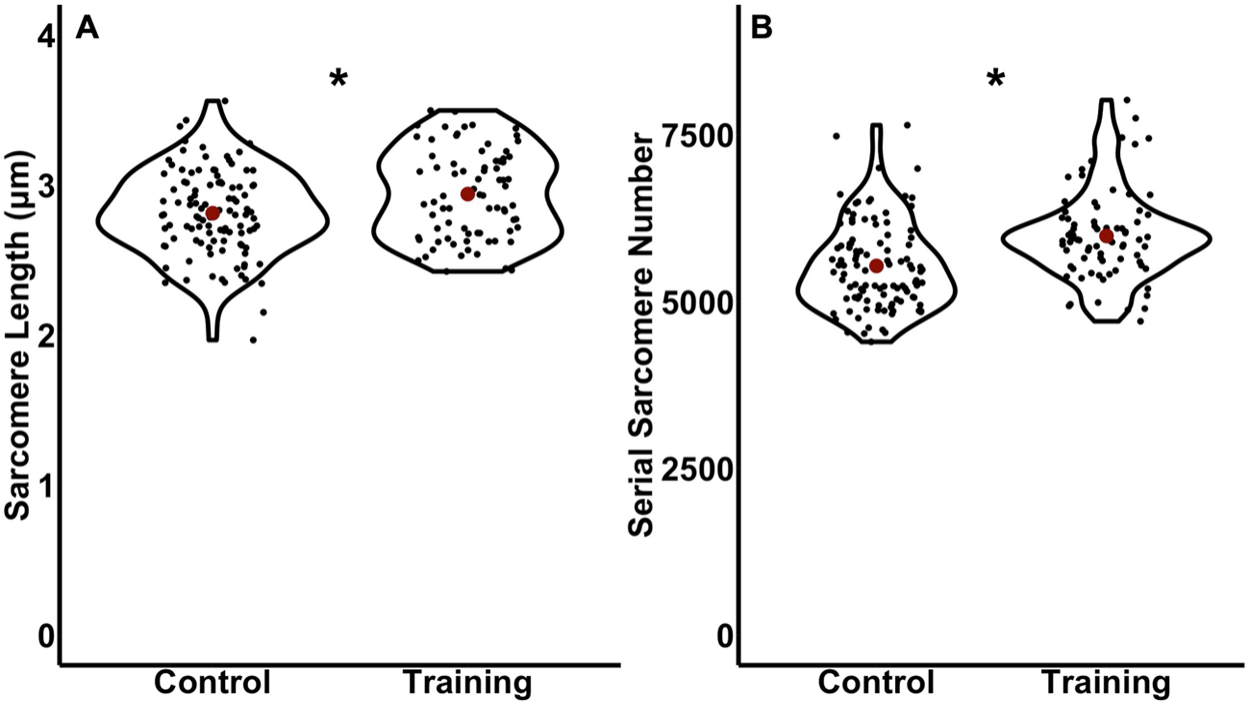
**Violin plot comparisons of average sarcomere length (A) and serial sarcomere number (B) in control versus trained rats with the sample size adjusted to treat each fascicle independently.** Red dots represent the mean. *Significant difference (*P*<0.05) between control and training.

For sarcomere length (SL), 80% power was not achieved using average SL in 18 control and 13 trained rats (Power=35%). With these sample sizes, SL did not differ (*P*=0.20) between trained [2.93±0.25 μm, 95% CI (2.78,3.07)] and control rats [2.81±0.25 μm, 95% CI (2.69,2.94)] (Fig. 3B). A follow-up a-priori power analysis indicated 67 control and 49 trained rats would be required to achieve statistical power for SL, which would not be feasible within time and ethical constraints of this research. To obtain data closer to this sample size, we treated each fascicle independently knowing that SLs are non-uniform and regionally variable throughout a muscle (Butterfield and Herzog, 2006; Moo et al., 2016), amounting to 106 control and 78 trained measurements of average SL. Viewed this way, SL was 5% longer (*P*<0.01, *d*=0.44) in trained [2.94±0.29 μm, 95% CI (2.87, 3.01)] than control rats [2.81±0.29 μm, 95% CI (2.76, 2.87)] (Fig. 4A). Therefore, there is evidence that training increased SL, however, only when using a larger sample size representative of individual fascicles as opposed to individual animals.

For average SSN, 82% power was achieved with 18 control and 13 trained rats. SSN was 8% greater (*P*=0.01, *d*=0.99) in trained [6024.45±473.38, 95% CI (5738.39, 6310.51)] compared to control rats [5577.03±464.52 mm, 95% CI (5346.03,5808.03)] (Fig. 3C). Fig. 4B also shows that SSN was 8% greater in trained compared to control rats when viewing the measurements from all fascicles (106 control and 78 trained fascicles) (*P*<0.01, *d*=0.69).

### Force-length relationships

**Fig. 5.**
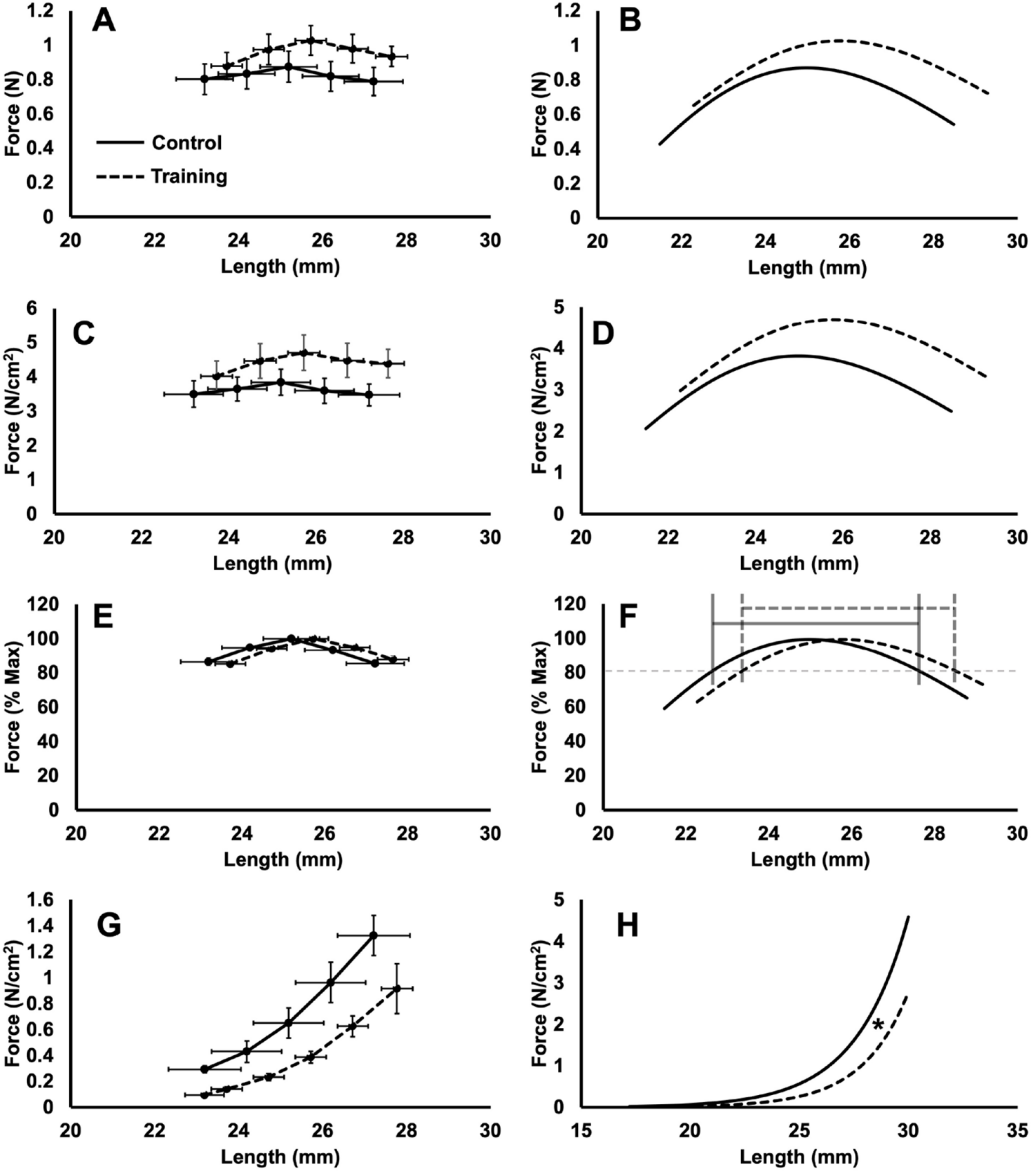
**Comparison of active force-length relationships in absolute terms (A,B), normalised to physiological cross-sectional area (C,D), and normalised to maximum force (E,F), and passive force-length relationships (G,H) in control (solid lines) versus trained rats (dashed lines), with the collected data on the left, and the corresponding fitted asymmetric Gaussian (B,D,F) and exponential curves (H) on the right.** The grey lines in F indicate the width of the plateau region. Data are reported as mean±s.e. (*n*=16 control, *n*=12 training). There were no between-group differences in optimal muscle length, maximum absolute or specific force, or the width of the plateau region. *The coefficient *b* indicated a less steep curve in trained compared to control rats (*P*<0.05).

The original data collected for the active force-length relationships are presented in Fig. 5A,C, and E, and the fitted curves are presented in Fig. 5B,D, and F. While visually the active force-length relationship of trained rats appears shifted to the right with greater forces than controls, there were no differences in L_O_ (trained: 25.78±1.27 mm; control: 24.98±3.05 mm; *P=*0.34), nor maximum absolute (trained: 1.03±0.30 N; control: 0.87±0.44 N; *P*=0.30) or specific force (trained: 4.69±1.82 N/cm^2^; control: 3.81±1.74 N/cm^2^; *P*=0.20). There were also no between-group differences in the width of the force-length relationship at 80% maximum (trained: 5.10±1.34 mm; control: 5.21±1.14 mm; *P=*0.82), suggesting similar widths of the plateau region. Altogether, these findings indicate the active force-length relationship was not different between trained and control rats.

The original data collected for the passive force-length relationships are presented in Fig. 5G, and the fitted curves are presented in Fig. 5H. There was no between-group difference in L_p_ (trained: 19.02±1.15 mm; control: 17.20±3.92 mm; *P*=0.13), but the coefficient *b* was 13% greater in trained rats (trained: 0.47±0.08 mm; control: 0.42±0.07 mm; *P*=0.05, *d*=0.76), indicating a less steep passive force-length curve and lower overall stiffness. These data indicate the passive force-length relationship of trained compared to control rats was not shifted to the right, but was less steep, resulting in reduced passive tension with stretch.

### Passive work loops

**Fig. 6.**
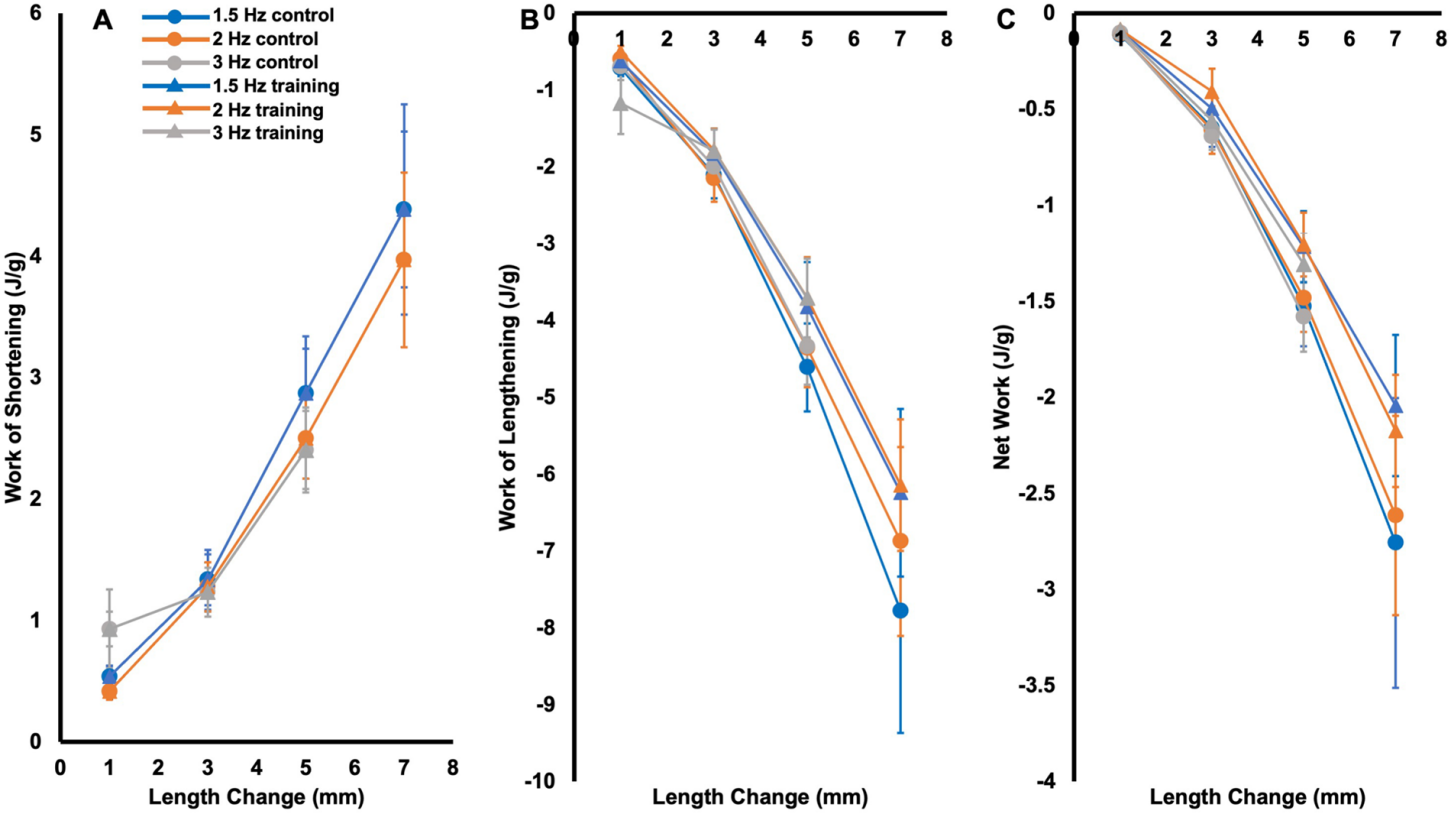
**Work of shortening (A), work of lengthening (B), and net work output (C) of the passive (i.e. no stimulation) work loops in trained and control rats**. Data are reported as mean±s.e. (*n*=16 control, *n*=12 training).

All passive work loops were clockwise in shape such that work of lengthening exceeded work of shortening (Fig. S2). There was an effect of group on net work output in the passive work loops [*F*(1304)=5.06, *P*=0.03, η_p_^2^=0.02], with trained rats producing 0.22 J/g less negative net work than controls across all work loops [95% CI of the difference (0.02,0.43 J/g)]. However, post-hoc *t*-tests did not reveal between-group differences in net work output in any specific passive work loops; hence, this difference was only noticeable when viewing all work loops together. There were no effects of group on work of shortening [*F*(1304)=1.12, *P*=0.29] or work of lengthening [*F*(1304)=3.08, *P*=0.08] in the passive work loops, and there were no interactions of group×cycle frequency×length change [*F*(5304)=0.06–0.09, *P*=0.92–1.00], group×cycle frequency [*F*(2304)=0.03–0.15, *P*=0.86–0.97], nor group×length change [*F*(3304)=0.32–1.02, *P*=0.38–0.81] on any passive work loop parameters (Fig. 6).

### Active work loops

**Fig. 7.**
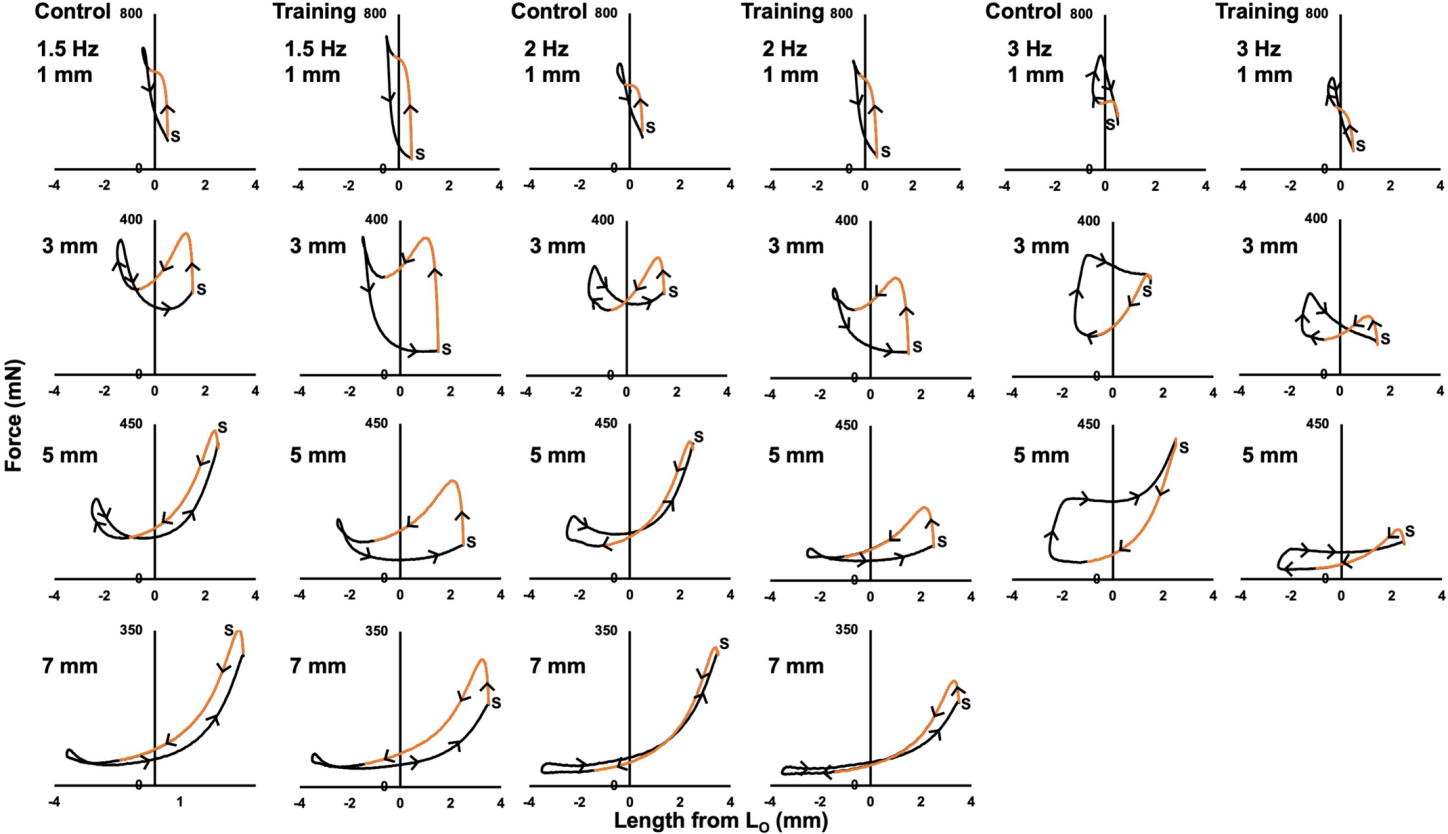
**Representative active (i.e. stimulation during shortening) work loop traces from 1 control and 1 trained rat.** *S* indicates the start of the cycle. Orange lines indicate the stimulation period. Arrows indicate the direction of the cycle, with clockwise segments containing negative work and counterclockwise segments containing positive work.

Fig. 7 shows representative work loop traces from one control and one trained rat. In general, work loops were mostly counterclockwise (i.e. containing primarily positive net work) up to work loops of at most 2 Hz and 3 mm, after which work loops became increasingly clockwise (i.e. containing negative work) with the work to re-lengthen the muscle exceeding the work of shortening.

**Fig. 8.**
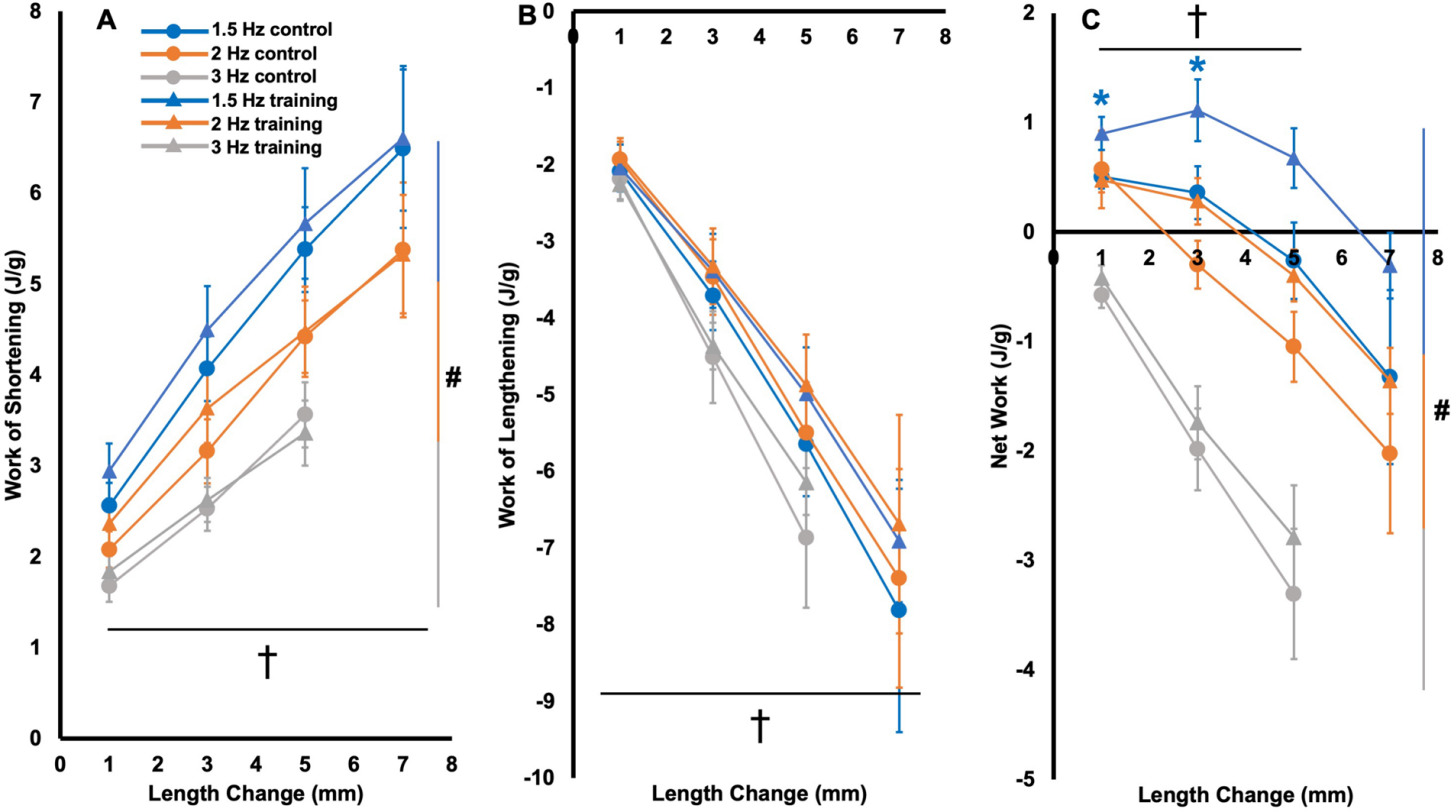
**Work of shortening (A), work of lengthening (B), and net work output (C) of the active (i.e. stimulation during shortening) work loops in trained (triangles) and control rats (circles).** Data are reported as mean±s.e (*n*=16 control, *n*=12 training). *Significant difference (*P*<0.05) between control and training at the colour-coded cycle frequency. #Significant difference across cycle frequencies. †Significant difference across length changes.

As shown in Fig. 8C, control rats on average produced maximal net work in the 1.5-Hz cycle at a 1-mm length change while trained rats produced maximal net work at 1.5 Hz and 3 mm, demonstrating a training-induced shift in the optimal length change at 1.5 Hz. Supporting training-induced changes in net work output, there was an effect of group on net work output [*F*(1304)=10.43, *P*<0.01, η_p_^2^=0.04], with trained rats producing on average 0.53 J/g [95% CI of the difference (0.21, 0.85)] more net work than controls across all active work loops. However, there were no interactions of group×cycle frequency×length change [*F*(5304)=0.06 *P*=1.00], group×cycle frequency [*F*(2304)=0.55, *P*=0.58], or group×length change [*F*(3304)=0.71, *P*=0.55], indicating the effect of group on net work output did not differ depending on cycle frequency and length change. Post-hoc *t*-tests showed the effect of group was most pronounced in 1.5-Hz work loops, with trained rats producing 78% greater net work output than controls at the 1-mm length change (*P*=0.04, *d*=0.83) and 209% greater net work output at the 3-mm length change (*P*=0.05, *d*=0.78) (Fig. 8C).

There were no effects of group on work of shortening [*F*(1304)=0.66, *P*=0.42] (Fig. 8A) or work of lengthening [*F*(1304)=1.58 *P*=0.21] in the active work loops (Fig. 8B). Neither work of shortening or work of lengthening showed interactions of group×cycle frequency×length change [*F*(5304)=0.01–0.02 *P*=1.00], group×cycle frequency [*F*(2304)=0.01–0.25, *P*=0.78– 0.99], or group×length change [*F*(3304)=0.20–0.32, *P*=0.81–0.89].

There were also effects of cycle frequency on work of shortening [*F*(2304)=21.44, *P*<0.01, η_p_^2^=0.13] and net work output, [*F*(2304)=64.79, *P*<0.01, η_p_^2^=0.32], but not work of lengthening [*F*(2304)=2.58, *P*=0.06]. There were effects of length change on all these variables [*F*(3304)=29.34–46.90, *P*<0.01, η_p_^2^=0.24–0.33]. Specifically, work of shortening decreased with increasing cycle frequency (all comparisons *P<*0.01), and work of shortening and work of lengthening both increased with length change (all comparisons *P*<0.01) (Fig. 8A,B). Net work output decreased with increasing cycle frequency (all comparisons *P*<0.01), and with increasing length change past the optimal length change, except for between 5 mm and 7 mm (*P=*1.00; all other comparisons *P*<0.01 to 0.03) (Fig. 8C).

**Fig. 9.**
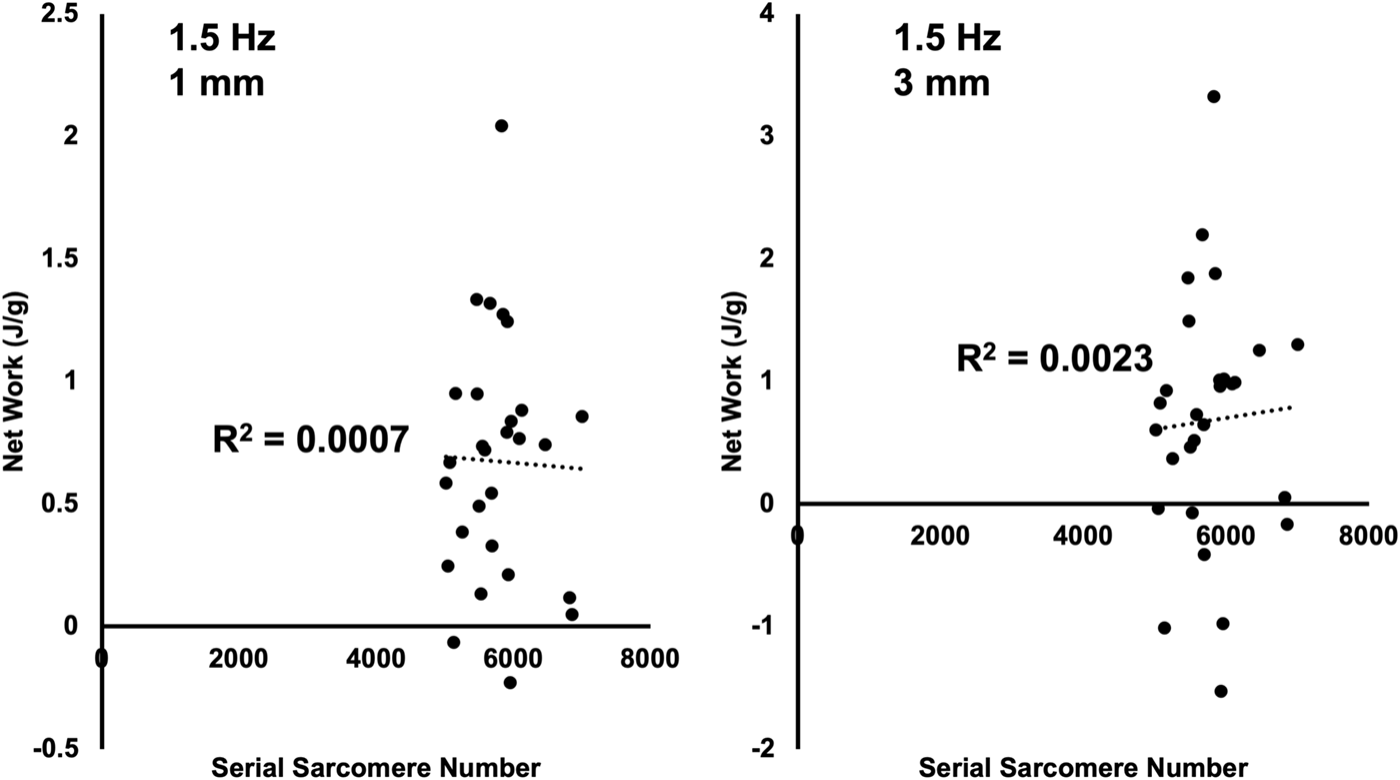
**Plots of the relationships between net work output and serial sarcomere number in the 1.5-Hz work loops at length changes of 1 and 3 mm**. There were no significant relationships between serial sarcomere number and net work output in these work loops (*P*>0.05).

Linear regressions were employed to investigate which variables may have contributed to the between-group differences in net work output in the 1.5 Hz work loops at 1 and 3 mm. There were no relationships between SSN and net work output in either work loop [1 mm: *F*(1,26)=0.02, *R*^2^<0.01, *P*=0.89; 3 mm: *F*(1,26)=0.06, *R*^2^<0.01, *P*=0.81] (Fig. 9), suggesting the training-induced increase in SSN contributed little to the improved work loop performance. There were also no relationships between net work output and the coefficient *b* (i.e. overall nonlinear stiffness of the passive force-length relationship) [1 mm: *F*(1,26)=2.00, *R*^2^=0.07, *P*=0.17; 3 mm: *F*(1,26)=0.86, *R*^2^=0.03, *P*=0.86], indicating the training-induced reduction in passive tension also had little effect on work loop performance. Absolute maximum isometric force was related to net work output in the 1.5-Hz, 1-mm work loop [*F*(1,26)=6.96, R^2^=0.21, *P*=0.01] but not the 3-mm work loop [*F*(1,26)=1.51, *R*^2^=0.06, *P*=0.23]. Specific maximum isometric force, however, was related to net work output in both work loops [1 mm: *F*(1,26)=18.66, *R*^2^=0.42, *P*<0.01; 3 mm: *F*(1,26)=5.20, *R*^2^=0.17, *P*=0.03]. Altogether, adaptations in maximum specific force likely contributed most to the improved work loop performance.

## DISCUSSION

This study assessed rat soleus architecture following 4 weeks of weighted downhill running training and aimed to relate muscle architectural adaptations to changes in dynamic contractile function, namely work loop performance. Our hypothesis that training would increase SSN, not change collagen content and crosslinking, and increase net work output was partly correct. Comparing trained and control rats, FL and SSN increased with training, as did SL but only when viewing every fascicle independently to increase the sample size. There were no differences in collagen parameters between groups, and increases in net work output were most pronounced in 1.5-Hz work loops at length changes of 1 and 3 mm.

### Did weighted downhill running training induce sarcomerogenesis?

The FL, SL, and SSN values in this study are within ranges of those recorded previously for the rat soleus (Aoki et al., 2009; Baker and Hall-Craggs, 1978; Chen et al., 2020; Jakubiec-Puka and Carraro, 1991; Koh and Tidball, 1999). The FL values being ∼60–70% of whole-muscle L_O_ is consistent with previous reports (Chen et al., 2020) and can likely be attributed to the digested fascicles no longer possessing connective tissue. It should also be noted that we only characterised global SSN of the soleus, thus any regional differences in SSN adaptations (Butterfield and Herzog, 2006) are beyond the scope of this study.

We observed an 8% training-induced increase in soleus SSN. This magnitude of sarcomerogenesis is greater than what Chen et al. (2020) observed following body weight downhill running compared to controls (an insignificant +3%). Butterfield and Herzog (2006) showed the magnitude of SSN increase is strongly related to peak force developed during eccentric contractions. Therefore, compared to Chen et al. (2020), the weighted vests employed in the present study may have enhanced the eccentric load while running downhill, resulting in a greater stimulus for sarcomerogenesis. In the present study, rats also ran 3 days/week (Monday, Wednesday, Friday) rather than the 5 days/week (Monday–Friday) used by Chen et al. (2020), and the extra recovery days may have allowed more time for remodelling and recovery between exercise bouts (Hyldahl and Hubal, 2014).

The most accepted hypothesis for sarcomerogenesis (i.e. an increase in SSN) stems from the active force-length relationship of muscle, whereby active force production is suboptimal in over-stretched positions due to limited actin-myosin binding (Gordon et al., 1966a,b), and observations that a muscle's average SL operating range favours force production for particular task demands (Lieber and Ward, 2011; Lutz and Rome, 1994; Rome, 1994). It follows that if a muscle is repeatedly forced to operate with overstretched sarcomeres (i.e. with eccentric training), SSN would increase to maintain optimal actin-myosin overlap regions in that position (Herring et al., 1984; Koh, 1995; Williams and Goldspink, 1978). These adaptations relate to a muscle's ability to sense a change in tension (in this case increased tension at a long muscle length), then convert that mechanical signal into biochemical events regulating protein synthesis (Aoki et al., 2009; Bogomolovas et al., 2021; Franchi et al., 2018; Soares et al., 2007; Spletter et al., 2018).

The 13% FL increase we observed with weighted downhill running training appears to have been driven by increases in both SSN (+8%) and SL (+4–5%). The increase in SL may be interpreted in two ways. First, as optimal SL is generally believed to be constant within a species (Gokhin et al., 2014; Walker and Schrodt, 1974), the roles of increased SL and SSN in increasing FL are likely not mutually exclusive, but rather depend on the time course of adaptations (Herzog and Fontana, 2022; Zöllner et al., 2012). This perspective is illustrated by Barnett et al. (1980), who stretched the patagialis muscle of chickens continuously for 10 days. Twenty-four hours after the onset of stretching, the researchers observed a 40% increase in biceps brachii SL, however, SL decreased back to normal by 72 h, with instead an increase in SSN. It is possible in the present study that at the 4-week mark, sarcomeres were at the point of stretching (i.e. increasing SL), while at the same time new sarcomeres had been added.

On the other hand, as we measured SL at L_O_, we may have observed an increase in optimal SL, which could imply elongation of the myofilaments within the sarcomere (Gokhin et al., 2014). In this regard, it is difficult to compare to previous studies on sarcomerogenesis, as most fixed their muscles at a set joint angle rather than at L_O_ (Roy et al., 1982; Salzano et al., 2018; Tabary et al., 1972; Williams and Goldspink, 1978). These studies reported on average approximately the same or shorter SLs in the experimental compared to control muscles. Therefore, it is hypothetically possible that, had they fixed the muscles at L_O_ (i.e. a longer length compared to the control muscle), they would have observed a longer optimal SL. This perspective is somewhat supported by other studies that fixed muscles at L_O_. Shrager et al. (2002) induced emphysema in the lungs of rats, then 5 months later performed lung volume reduction surgery to treat the emphysema. Another 5 months later, the diaphragms of rats who received surgery had a 14% higher SSN and a 2% longer SL at L_O_ than untreated rats (2.95 µm versus 2.88 µm). Coutinho et al. (2004) fixed the rat soleus at resting length, which they assumed to be L_O_, following 3 weeks of immobilisation in a shortened position with intermittent stretching. Although they observed a decrease in SSN (due to being immobilised in a shortened position), they observed a ∼2% increase in SL compared to controls (2.2 µm versus 1.9 µm). Lastly, Chen et al. (2020) fixed the rat soleus at a measured L_O_ and observed on average a greater SL in downhill running rats than controls (2.77±0.09 µm versus 2.75±0.11 µm). Future research might aim to assess the time course of SL and SSN adaptations during training or confirm optimal SLs via single-fibre testing to better understand these observations.

### Did sarcomerogenesis impact the force-length relationship?

We observed on average a 3% greater L_O_ in trained than control rats, however, it was not statistically significant due to high variability, particularly in the control group (Fig. 5). With an increase in SSN, L_O_ is expected to shift to a muscle length previously on the descending limb (Davis et al., 2020; Koh, 1995). Rightward shifts in L_O_ of 5–12% have been observed alongside 7–20% increases in SSN in studies on animals via immobilisation in a stretched position, high-acceleration training, isokinetic eccentric training, and downhill running training (Butterfield and Herzog, 2006; Lynn et al., 1998; Salzano et al., 2018; Williams and Goldspink, 1978). The downhill running study (Lynn et al., 1998) assessed the joint torque-angle (i.e. not muscle force-length) relationship of the vastus intermedius, so is difficult to compare to the present study. Chen et al. (2020), though, employed downhill running training and observed no difference in rat soleus L_O_ compared to controls, and even compared to uphill running (i.e. decreased SSN) rats, on average only a 1.5% (also insignificant) greater L_O_. Therefore, like with SSN, the present study's addition of weighted vests and extra recovery days between training sessions may have amounted to a greater shift in rat soleus L_O_ compared to body weight downhill running training, even if it was statistically insignificant.

Changes to the passive force-length relationship were more noticeable than changes to the active force-length relationship. Though there was no apparent shift in L_p_, trained rats had a 13% greater *b* coefficient, indicating a less steep passive force-length curve and lower overall stiffness (Fig. 5H). This adaptation in passive tension also manifested in the passive work loops, with an effect of group showing 0.22 J/g less negative net work output in trained rats across all passive work loops. With a greater SSN, individual sarcomeres may stretch less during muscle excursion, leading to less passive force generated by sarcomeric proteins such as titin (Herbert and Gandevia, 2019). Noonan et al. (2020) demonstrated this with lower passive stress and passive elastic modulus in soleus single fibres of rats that ran downhill compared to uphill. Noonan et al. (2020) used the same rats as Chen et al. (2020), therefore these lower passive properties appeared to be due to a 6% greater SSN.

The influence of sarcomerogenesis on the passive length-tension relationship is not always predictable due to the concurrent involvement of intramuscular collagen in passive force generation (Gillies and Lieber, 2011). For example, in studies on animals with sarcomerogenesis induced via surgically stretching a muscle, the passive force-length relationship steepens due to concurrent increases in collagen content (Herbert and Balnave, 1993; Takahashi et al., 2012). Age-related increases in passive muscles stiffness are also related to increased collagen crosslinking in mice (Brashear et al., 2020). We observed no differences in collagen content or crosslinking between trained and control rats, with our values of rat soleus hydroxyproline concentration falling into ranges of previous studies (Karpakka et al., 1992; Sugama et al., 1999). Han et al. (1999) observed no change in collagen content in the rat soleus following downhill running, but also observed increases in some mRNAs and enzymes related to collagen synthesis; however, these were following only a single bout of exercise. Perhaps more comparable to the present study, Zimmerman et al. (1993) observed no changes in soleus collagen content or crosslinking in 20-week-old rats following 10 weeks of uphill running training. A less steep passive force-length relationship alongside increased SSN and no change in collagen content was also observed following passive stretch training of the rabbit soleus (De Jaeger et al., 2015), aligning with our findings.

### Is the work loop data comparable to previous studies on the rat soleus?

The present study's active work loops employed stimulation duty cycles of 0.7, which is longer than optimal for all the cycle frequencies used (optimal=∼0.6 at 1.5 Hz, ∼0.55 at 2 Hz, and ∼0.45 at 3 Hz; Swoap et al., 1997). Consequently, full relaxation did not occur prior to the lengthening phase in many of the active work loops, leading to some active lengthening force development and partly, if not fully (i.e. at 3 Hz), clockwise work loops (Fig. 7). Comparisons with previous work loop studies on the rat soleus are then difficult, however, the work loops that were counter-clockwise (e.g. Fig. 7: Training, 2 Hz, 5 mm) were similar in shape to previous representative traces for the rat soleus (Swoap et al., 1997). Additionally, the general shapes of the net work×length change×cycle frequency graphs (Fig. 8C), showing net work output decreases with increasing cycle frequency and on either side of the optimal length change, are similar to previous reports (Caiozzo and Baldwin, 1997; Swoap et al., 1997). Therefore, the influence of varying length change and cycle frequency on work loop performance was maintained regardless of the nonideal cycle frequency. Although no effect of cycle frequency was observed on work of lengthening in the active work loops, since full relaxation did not occur prior to lengthening, it is also likely that the drop in net work output with increasing cycle frequency was further enhanced than normal, as there would be increasingly greater eccentric force (i.e. active force) development, given that eccentric force increases with lengthening velocity (Alcazar et al., 2019).

Swoap et al. (1997) determined optimal length change for the rat soleus at 1.5 Hz to be 5 mm, while in the present study it was 1 to 3 mm. In addition to the shorter stimulation duty cycle used, this may be because Swoap et al. (1997) tied the muscle at the distal end of the Achilles tendon, while we tied at the musculotendinous junctions. Incorporating tendon into work loops can increase net work output by allowing muscle fibres to shorten at closer to optimal speeds (Lichtwark and Barclay, 2010). Swoap et al. (1997) also used an *in situ* setup with an intact blood supply (unlike our *in vitro* setup), which can enhance force production, and used a higher temperature (30°C versus 26°C), which may permit better force-generating ability during faster and longer excursion contractions (Ranatunga, 2018; Roots and Ranatunga, 2008). Altogether, while difficult to compare to previous studies, the overall function of the rat soleus across a range of work loop cycle frequencies and length changes are consistent with previous reports.

### Did sarcomerogenesis relate to improvements in work loop performance?

We hypothesised that a longer FL and greater SSN induced by weighted downhill running would improve work loop performance, particularly at faster cycle frequencies and longer length changes. An effect of group showed that trained rats produced overall greater net work output in the active work loops, with the most pronounced increases occurring in the 1.5-Hz loops at 1 and 3 mm (Fig. 8C). Training-induced sarcomerogenesis may not have contributed to these improvements in work loop performance, however. If sarcomerogenesis brought force production closer to optimal across a wider range of muscle lengths, we would have seen a widening of the force-length relationship's plateau region (Akagi et al., 2020), which we did not observe (Fig. 5F). Additionally, net work output in these work loops did not relate to SSN (Fig. 9), and related more strongly to specific maximum isometric force. While there were no significant training-induced increases in specific maximum isometric force, the forces of trained rats appear visually higher (Fig. 5D) and a significant difference may not have been observed due to high variability (Fig. 5C). Increased specific force following downhill running training was also observed in rat soleus single fibres (Mashouri et al., 2021). Increased specific force production has been attributed to increased myosin concentration and increased packing density of contractile filaments (Parente et al., 2008; Penman, 1970), while others have argued against this, saying the mechanisms are unknown (Pansarasa et al., 2009). Regardless, it appears that training-induced adaptations in the muscle's intrinsic force-generating capacity contributed most to the improved work loop performance.

Our hypothesis may also only stand if SL at L_O_ remained the same between control and trained rats. With increased SSN but the same SL, sarcomeres would stay in closer range to optimal SL, and shortening velocity of individual sarcomeres would be reduced for a given muscle excursion, improving active force production and thereby the mechanical work (Baxter et al., 2018; Drazan et al., 2019). We observed a 4–5% increase in SL at L_O_ with training, so the sarcomere shortening velocities and operating ranges with respect to optimal SL may not have changed.

In Table 1, we estimated the sarcomere excursions relative to optimal SL and sarcomere shortening velocities in the 1.5-Hz work loops at length changes of 1 and 3 mm in trained and control rats. To do this, we first determined the average fraction of the measured FLs compared to the whole-muscle L_O_ (0.65). By doing this, we can estimate what the FL change would be for a given whole-muscle excursion (i.e. a whole-muscle excursion of ±0.5 mm would be ±0.5 mm×0.65=±0.325 mm; a whole-muscle excursion of ±1.5 mm would be ±1.5 mm×0.65=±0.975 mm). Subsequently, we can estimate the SL at that FL by dividing FL by the SSN [e.g. at L_O_+1.5 mm, (17.523 mm+0.975 mm)/6024=3.07 μm; at L_O_ – 1.5 mm, (17.523 mm – 0.975 mm)/6024=2.75 μm]. Assuming the width of the sarcomere force-length relationship changed proportionally to SL, we can calculate the SL excursion relative to SL at L_O_ during the whole-muscle excursion {e.g. [(3.07–2.75 μm)/2.93]×100%=10.92%}. We can also estimate the sarcomere shortening velocity in an excursion by multiplying the sarcomere displacement by 2 times the cycle frequency [e.g. (3.07–2.75 μm)×(1.5 Hz×2)=0.96 μm/s]. Because SL at L_O_ is different between groups, these velocities should also be expressed as SL/s for comparisons (e.g. 0.96 μm/s/2.93 μm=0.33 SL/s).

**Table 1.**
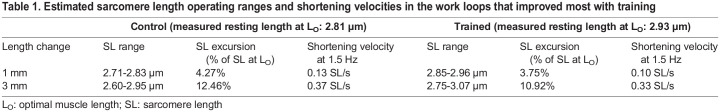
Estimated sarcomere length operating ranges and shortening velocities in the work loops that improved most with training

From the estimations in Table 1, the sarcomeres of trained rats could conceivably shorten/lengthen relatively less with respect to optimal SL and operate at relatively slower shortening velocities than controls, placing sarcomeres at more advantageous positions on the force-length and force-velocity relationships, and contributing to greater net work output. However, differences in sarcomere excursions and shortening velocity of 0.52–1.54% and 0.03–0.04 SL/s, respectively, are small, making them subject to error and perhaps irrelevant when scaled to the whole muscle due to series compliance (Kawakami and Lieber, 2000). Additionally, as shown in Fig. S3A,B, if SL at L_O_ had been constant between trained and control rats [whether to achieve the observed SSN (A) or FL (B)], the trained values still would not have been much less than control values. In Fig. S3C, we also estimated whether a hypothetically very large magnitude of sarcomerogenesis [e.g. a 33% SSN increase observed by Williams (1990) from stretch training following immobilisation in a shortened position] would alter these findings, and found that this SSN increase may produce much larger reductions in relative sarcomere excursion and sarcomere shortening velocity compared to controls. Altogether, while the calculations in Table 1 prove the feasibility of our hypothesis, it is unlikely that sarcomerogenesis contributed to improved work loop performance in the present study, and a very large magnitude of sarcomerogenesis may be needed for SSN to influence work loop performance.

To our knowledge, one other study on animals has assessed the impact of sarcomerogenesis on work loop performance (Cox et al., 2000). After incrementally surgically stretching the rabbit latissimus dorsi for 3 weeks, they observed a 25% increase in SSN, however, maximum work loop power output decreased by 40%. They determined this detriment to work loop performance was related to energy loss (i.e. more negative work in passive work loops) caused by collagen accumulation, because after an additional 3 weeks of maintained stretch, collagen content decreased back toward control values, SSN increased another 5%, and work loop power output returned to normal. As the present study observed no changes in collagen content or crosslinking per unit volume of muscle, and an overall improvement (i.e. effect of group) in performance across all work loops, we were able to demonstrate that work loop performance is not impaired when intramuscular collagen concentration and crosslinking remains the same.

### Limitations

We used the rat soleus in work loop experiments, rather than the more commonly used mouse soleus (Hill et al., 2019), which, due to its smaller size, is better able to retain tissue viability. However, we took several precautions to ensure the viability of the rat soleus during our experimental protocols. While data collection at closer to physiological temperature (37°C) would have permitted better real-world applicability of our data, we used a lower temperature (26°C) to slow deterioration of the muscle tissue, which has been shown to be effective for rat soleus *in vitro* experiments (Barclay, 2005). Close to this temperature (27°C) was also used previously for long-duration *in vitro* experiments on the rat soleus in our lab and was found to maintain of the health of the muscle preparation (Chen et al., 2020). Additionally, to minimise time spent on the force-length relationship prior to work loop protocols, the active and passive force-length relationships were only constructed over a small range of muscle lengths (usually L_O_±2 mm) across 1-mm intervals. Though more in-depth collection of force-length data would have been ideal, curve-fitting allowed us to address this shortcoming to the best of our ability within our experimental design to preserve the health of the muscle preparations. We also only employed one passive work loop and two active work loops per set to minimise the amount of stretch and stimulation being imposed on the muscle throughout the experiment. There is likely some viscosity present during the first loop that would have been reduced in later cycles and yielded different net work output values, however, previous work loop studies on the rat soleus also employed as few as three total work loops per set in assessing net work output across a range of length changes and cycle frequencies (Swoap et al., 1997). While our efforts to preserve tissue viability presented other limitations, they were effective, as force and work generating capacity did not deplete from more than 20% and 2%, respectively throughout the experiment, which fall within previously used guidelines (James et al., 1996).

As discussed earlier, our study used a non-ideal duty cycle of 0.7, which did not permit full relaxation of the muscle prior to the lengthening phase of the work loops, especially at the 2 and 3-Hz cycle frequencies. While this limited our ability to compared to previous work loop data from the rat soleus (Caiozzo and Baldwin, 1997; Swoap et al., 1997), these conditions were consistent between the control and trained muscles, and therefore were unlikely to interfere with our between-group comparisons of work loop data. It is of course possible that our comparisons may have differed if optimal duty cycles were employed; however, as the present study's hypothesis relied on force data collected during dynamic muscle length changes, and not stimulation duration, we can speculate that optimal duty cycles would have only yielded greater net work values for both control and trained rats with similar between-group differences.

### Conclusion

The purpose of this study was to assess muscle architecture and work loop performance of the rat soleus following 4 weeks of progressively loaded weighted downhill running training. Aligning with our hypotheses, longitudinal muscle growth occurred, with a 13% increase in FL that appeared to stem from an 8% increase in SSN and a 4–5% increase in SL. This longitudinal muscle growth corresponded to a less steep passive force-length curve, and a more rightward-shifted (though still insignificant) active force-length relationship compared to previous body weight downhill running training. Work loop performance improved most at the slowest cycle frequency with the two shortest length changes. While there were improvements in work loop performance, it did not appear to be dependent on the observed sarcomerogenesis, possibly because the magnitude of sarcomerogenesis was not large enough to impact dynamic contractile function at the whole-muscle level.

## MATERIALS AND METHODS

### Animals

Thirty-one male Sprague-Dawley rats (euthanised at ∼18 weeks) were obtained (Charles River Laboratories, Senneville, QC, Canada) with approval from the University of Guelph's Animal Care Committee and all protocols following CCAC guidelines. Rats were housed at 23°C in groups of three and given *ad libitum* access to a Teklad global 18% protein rodent diet (Envigo, Huntington, Cambridgeshire, UK) and room-temperature water. After a week of acclimation to housing conditions and familiarisation with the vests and treadmills, rats were assigned to one of two groups: training (*n*=13) or control (*n*=18). Trained rats participated in 4 weeks of weighted downhill running 3 days/week, while control rats remained sedentary. Approximately 72 h following recovery from the final training day, rats were sacrificed via CO_2_ asphyxiation and cervical dislocation (Chen et al., 2020). We then immediately dissected the soleus and proceeded with mechanical testing. The soleus was chosen for this study due to its simple fusiform structure with fascicles running tendon to tendon (Williams and Goldspink, 1973), its expected lack of changes in collagen content and crosslinking (Han et al., 1999; Zimmerman et al., 1993), and its suitability for prolonged *in vitro* experiments, being a primarily slow-fibered muscle (Barclay, 2005; Caiozzo and Baldwin, 1997; Swoap et al., 1997). Due to measurement errors in characterising the force-length relationship after determining L_O_, two rats (one control, one training) were excluded from analysis of the force-length relationship. Similarly, due to a faulty setup of work loop protocols, the same two rats plus one other (two control, one training) were excluded from work loop data analyses.

### Weighted vests

After extensive piloting, we designed a custom-made weighted rodent vest that is both well tolerated by the rat and sufficiently adds weight during running (Fig. 1). A child-sized sock was cut into the shape of a vest: the end of the sock was cut off such that the ankle end went around the rat's neck and the toe end went around the rat's torso, then arm holes were cut on each side just distal to the neck hole. A T-shaped piece of foam paper was then super-glued on the back of the vest starting above the arm holes. A piece of Velcro was then placed across the wider section of the T-shaped foam paper. The apparatus for holding the weights was then constructed. A strip of packing tape was cut to be the same length as the wider section of the T-shaped foam paper. To allow for easier manipulation of the weight apparatus, a pipe cleaner of the same length was then cut and stuck across the midline of the tape, then a thinner piece of tape was placed over top to hold the pipe cleaner in place. Two 2 cm×2 cm Ziplock bags were then stuck on the strip of tape overtop of the pipe cleaner, equidistant from the middle. Additional small strips of tape were added as needed to secure these bags in place. A connecting piece of Velcro was then placed on the side of the tape opposite the bags, allowing it to be fastened to the T-shaped foam paper. The appropriate weights (various sizes of coins) were placed in the Ziplock bags. The bags were small enough to hold the coins securely in place such that they did not move around as the rat ran.

### Training program

The training program was modelled after those by Butterfield et al. (2005) and Chen et al. (2020), who saw SSN adaptations following 2–4 weeks of downhill running training in the rat vastus intermedius and soleus, respectively, compared to control and uphill running groups. Two weeks prior to training, rats were handled for 1 h on three consecutive days to reduce their stress levels when later applying the vests. The next week (i.e. 1 week before training), the rats underwent five consecutive days of familiarisation sessions on the treadmill, each consisting of three 3-min sets at a 0° grade and 10–12 m/min speed, with 2 min of rest between each set. In the first two familiarisation sessions they did not wear vests, in the third session they wore a non-weighted vest for 1/3 sets, in the fourth session they wore a non-weighted vest for 2/3 sets, and in the fifth session they wore a non-weighted vest for all 3 sets. Rats who were not compliant to the treadmill after 3 days of attempted familiarisation were made controls instead.

Previous models of eccentric-biased resistance training in rats, rabbits, and mice optimised muscle architectural adaptations with lower compared to higher training frequencies (Butterfield and Herzog, 2006; Morais et al., 2020; Ochi et al., 2007; Wong and Booth, 1988; Zhu et al., 2021), so rats ran 3 days per week (Monday, Wednesday, and Friday). Rats ran on an EXER 3/6 animal treadmill (Columbus Instruments, Columbus, OH, USA) set to a 15° decline. Rats ran in 5-min bouts, separated by 2 min of rest. They completed three bouts on the first day and increased by two bouts/day up to the seven-bout target (35 min total) on the third day of training, which persisted for the remainder of the training period. Rats began each training session at a speed of 10 m/min, which was increased by 1 m/min up to the 16 m/min target (Chen et al., 2020). Progressive loading was employed by adding weight equivalent to 5% of the rat's body mass during the first week, 10% in the second week, 15% in the third week, and 15% readjusted to the new body mass in the fourth week. The training efficacy and safety of this approach was modelled after a weighted vest training review that reported added weights of 5–20% body mass (Macadam et al., 2022), and work by Schilder et al. (2011), who added up to 36% of body mass to rats via backpacks to simulate obesity. 15% of body mass was the maximum weight used in the present study because during piloting we found that adding 20% of body mass was too cumbersome for the animals while running. Each training session took place at approximately the same time of day (between 9 and 11 AM).

### Experimental setup

Immediately following euthanasia, the soleus was carefully harvested from the right hindlimb (Ma and Irving, 2019). Silk-braided sutures (USP 2-0, metric 3) were tied along the musculotendinous junctions and mounted to the force transducer and length controller in the 806D Rat Apparatus (Aurora Scientific, Aurora, ON, Canada). The muscles were bathed in a ∼26°C Tyrode solution with a pH of ∼7.4 (121 mM NaCl, 24 mM NaHCO_3_, 5.5 mM D-Glucose, 5 mM KCl, 1.8 mM CaCl_2_, 0.5 mM MgCl_2_, 0.4 mM NaH_2_PO_4_, 0.1 mM EDTA) that was bubbled (Bonetta et al., 2015; Cheng and Westerblad, 2017) with a 95% O_2_/5% CO_2_ gas mixture (Praxair Canada Inc., Kitchener, ON, Canada). A 701C High-Powered, Bi-Phase Stimulator (Aurora Scientific, Aurora, ON, Canada) was used to evoke all contractions via two parallel platinum electrodes submerged in the solution, situated on either side of the muscle. Force, length, and stimulus trigger data were all sampled at 1000 Hz with a 605A Dynamic Muscle Data Acquisition and Analysis System (Aurora Scientific, Aurora, ON, Canada). All data were analysed with the 615A Dynamic Muscle Control and Analysis High Throughput (DMC/DMA-HT) software suite (Aurora Scientific, Aurora, ON, Canada).

### Mechanical testing

Mechanical testing of the soleus proceeded from Protocol A to D. As a starting point for approximating optimal length, the soleus was passively set to a taut length that resulted in ∼0.075 N of resting tension prior to beginning any protocols (Chen et al., 2020).

#### Protocol A: twitch current

Single 1.25-ms pulses were delivered in increasing increments of 0.5 mA (starting at 1 mA) until peak twitch force was elicited. The current for peak twitch force was used in the remainder of mechanical testing.

#### Protocol B: force-length relationships

The muscle was lengthened in 1-mm increments, with maximal tetanic stimulation (0.3 ms pulse width, 1 s duration, 100 Hz frequency) delivered at each new length (Chen et al., 2020; Heslinga and Huijing, 1993). This was repeated until the muscle length that produced peak tetanic force (L_O_) was reached. Construction of an active force-length relationship was then completed to at least ±2 mm with respect to L_O_. To further approximate/confirm L_O_ for the subsequent work loop experiments, the muscle was stimulated at the initial L_O_±0.5 mm. This final length for L_O_ was measured from tie to tie (i.e. musculotendinous junction to musculotendinous junction) using 150 mm (0.01 mm resolution) analogue callipers (Marathon, Vaughan, ON, Canada), and the muscles were set at this length prior to proceeding to the work loops. Prior to each tetanic stimulation, 2 min (to allow for dissipation of force transients owing to stress-relaxation) of resting force were obtained for construction of a passive force-length relationship, and following each tetanic stimulation, the muscle was returned to the original taut length (Heslinga and Huijing, 1993).

#### Protocol C: active and passive work loops

Sinusoidal muscle length changes of 1, 3, 5, and 7-mm (e.g. a 3-mm length change indicates a displacement of ±1.5 mm from L_O_) were imposed at cycle frequencies (i.e. the frequency of the length sinusoid) of 1.5, 2, and 3 Hz. After piloting, we decided to not test the 7-mm length change at the 3-Hz cycle frequency because this produced a large amount of negative work, and we wanted to minimise risk of damaging the muscle. The range of cycle frequencies was chosen because they correspond to a wide range of treadmill running speeds for the rat and are consistent with cycle frequencies that occur during spontaneous running (maximum 4 Hz) (Caiozzo and Baldwin, 1997; Nicolopoulos-Stournaras and Iles, 1984; Roy et al., 1991). The range of length changes was chosen because they fall within the maximum length change of the rat soleus *in vivo* (∼10 mm) (Woittiez et al., 1985). The cycle frequency and length change ranges we used are also comparable to those used in previous rat soleus work loop studies (Caiozzo and Baldwin, 1997; Swoap et al., 1997).

A set of three consecutive work loops was performed at each cycle frequency: one passive work loop with no electrical stimulation to determine the work done against passive elements in the muscle (Cox et al., 2000; James et al., 1995); and two active work loops with stimulation (0.3 ms pulse width, 100 Hz frequency) set to begin at the onset of shortening (Swoap et al., 1997) and last for 70% of the shortening half of the cycle (i.e. duty cycle=0.7). While a duty cycle of 0.7 did not allow full relaxation of the muscle prior to the lengthening half of the cycle, especially at the 2 and 3-Hz cycle frequencies (Swoap et al., 1997), as this duty cycle was consistent between trained and control rats, we were still able to address the present study's research question on training-induced work loop adaptations in relation to muscle architecture.

Pilot testing determined that two consecutive active work loops sufficiently determined optimal net work: in a set of three active work loops, the highest net work occurred in the first or second loop more than 80% of the time, and net work did not differ between each loop by >10%. Employing only two active work loops per set was also desirable for minimising the development of muscle fatigue and deterioration of tissue viability. Two minutes of rest were always provided before proceeding to the next set of work loops (Swoap et al., 1997).

#### Protocol D: assessment for fatigue-induced force loss

Two minutes after the final work loop, a maximal tetanic contraction at L_O_, and a work loop at 1.5 Hz and 3 mm (i.e. the loop determined to usually produce maximal net work output during piloting) were performed to ensure that fatigue did not interfere with results. Fatigue was defined as these parameters dropping below 80% of the baseline values (James et al., 1996), and no muscles experienced this.

### Muscle architecture and serial sarcomere number estimations

Following mechanical testing, the muscle was removed from the bath, weighed, then passively stretched to the final L_O_ determined in Protocol B and tied to a wooden stick. The muscle was then fixed in 10% phosphate-buffered formalin for ∼1 week, rinsed with phosphate-buffered saline, and digested in 30% nitric acid for 6–8 h to remove connective tissue and allow individual muscle fascicles to be teased out (Butterfield et al., 2005; Chen et al., 2020). To obtain a global measure of SSN, six fascicles were obtained lateral to medial, superficial, and deep across the muscle. Dissected fascicles were placed on glass microslides (VWR International, USA), then fascicle lengths were measured using ImageJ software (version 1.53f, National Institutes of Health, USA) from pictures captured by a level, tripod-mounted digital camera, with measurements calibrated to a ruler that was level with the fascicles. SL measurements were taken at six different locations along each fascicle via laser diffraction (Coherent, Santa Clara, CA, USA) with a 5-mW diode laser (25 μm beam diameter, 635 nm wavelength) and custom LabVIEW program (Version 2011, National Instruments, Austin, TX, USA) (Lieber et al., 1984), for a total of 36 SL measurements per muscle. Serial sarcomere numbers were calculated as:

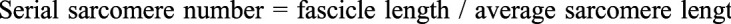


### Collagen content and solubility assay

The left soleus of 11 control and 12 trained rats was dissected and stored at −80°C until use in a hydroxyproline with collagen solubility assay, with pepsin-insoluble collagen quantifying the amount of collagen crosslinking (Brashear et al., 2020). The muscle was powdered in liquid nitrogen with a mortar and pestle, careful to remove any remaining tendon. The powdered muscle was then weighed and washed in 1 ml of phosphate-buffered saline and stirred for 30 min at 4°C. Non-crosslinked collagen was digested in a 1:10 mass (mg of powdered tissue) to volume (µl) solution of 0.5 M acetic acid with 1 mg/ml pepsin, stirring overnight at 4°C. After centrifuging at 16,000 ***g*** for 30 min at 4°C, the supernatant was collected as the pepsin-soluble fraction (non-crosslinked collagen), and the pellet was kept as the pepsin-insoluble fraction (crosslinked collagen). The two separate fractions were hydrolysed overnight in 0.5 ml of 6 M hydrochloric acid at 105°C. 10 µl of hydrolysate were mixed with 150 µl of isopropanol followed by 75 µl of 1.4% chloramine-T (Thermo Fisher Scientific) in citrate buffer (pH=6.0) and oxidised at room temperature for 10 min. The samples were then mixed with 1 ml of a 3:13 solution of Ehrlich's reagent [1.5 g of 4-(dimethylamino) benzaldehyde (Thermo Fisher Scientific); 5 ml ethanol; 337 µl sulfuric acid] to isopropanol and incubated for 30 min at 58°C. Quantification was determined by extinction measurement of the resulting solution at 550 nm. A standard curve (0-1000 µM trans-4-hydroxy-L-proline; Thermo Fisher Scientific) was included in each assay (average linear R^2^=0.98), and samples were run in triplicate (average CV=0.098). Hydroxyproline concentrations in samples were determined by interpolation from the linear equation of the corresponding assay's standard curve. Results are reported as µg of hydroxyproline per mg of powdered tissue mass.

### Data and statistical analyses

All work values were normalised to muscle wet weight (g). Active and passive force values were normalised to the muscle's physiological cross-sectional area (PSCA; cm^2^) to obtain specific values (expressed as units/cm^2^). PCSA was calculated as:

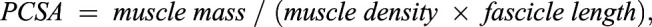
with muscle density assumed to be 1.112 g/cm^3^ (Ward and Lieber, 2005).

For construction of active force-length relationships, passive force values were subtracted from total force at each length to estimate active force production. Passive force was taken from a 500-ms window prior to tetanic stimulation, and total force was taken as the maximum force produced in the tetanic contraction. From the collected data, average active and passive force-length relationships for each group were constructed by averaging force values in length categories with respect to L_O_ (e.g. L_O_ −2, −1, 0, +1, +2 mm). The passive force-length relationship was plotted with force in units of N/cm^2^ (Binder-Markey et al., 2021) and the active force-length relationship was plotted in units of N, N/cm^2^ and % maximum force. Since these data were collected in 1-mm intervals, they were fitted to curves using least square optimisation (Microsoft Excel version 16.60 Solver add-on). Active force-length relationships were fitted to an asymmetric Gaussian function (Mohammed and Hou, 2016):

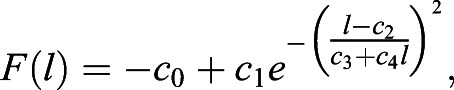
with −*c*_0_ shifting the function down, *−c*_0_ *+ c*_1_ representing the maximum isometric force, *c*_2_ representing L_O_, and *c*_3_ and *c*_4_ being associated with the width of the function on both sides of L_O_. The fitted active force-length relationships were constructed in absolute terms (units of N), normalised to PCSA, and normalised to maximum force, and plotted up to 3.5 mm on either side of L_O_ (to match the maximum excursion of the work loops). From the fitted curves, two-tailed *t*-tests were used for between-group comparisons of L_O_, as well as the width of the curve at 80% maximum force (Akagi et al., 2020; Lynn et al., 1998) to investigate the width of the plateau region.

Per recommendations from the systematic review by Binder-Markey et al. (2021), passive force-length relationships were fitted to an exponential function. We used the exponential function employed by Gollapudi and Lin (2009) from modelling by Zajac (1989) to allow computation of both the overall nonlinear stiffness coefficient *b*, and slack length (L_p_):

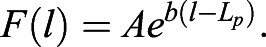


From the fitted exponential curves, two-tailed *t*-tests were used for between-group comparisons of L_p_ and *b*.

From each work loop, Aurora Scientific software calculated work of shortening and work of lengthening (the integrals under the shortening and lengthening curves, respectively, in a force-length plot, with work in the shortening direction defined as positive) and net work output of a whole cycle (the area inside the whole shortening and lengthening cycle, with a counterclockwise loop defined as positive). Of the two active work loops in a given set, the loop that produced the highest net work was chosen for statistical analyses (Swoap et al., 1997).

All the following statistical analyses were conducted in IBM SPSS statistics version 26. Two-tailed *t*-tests were used to compare body weights between control and trained rats throughout the training period (i.e. at ∼14, 15, 16, 17, and 18 weeks old), and to compare FL, SL, SSN, muscle wet weight, PCSA, total collagen, and % insoluble collagen between trained and control rats. Since characterisation of longitudinal muscle growth was the primary objective of this study, post hoc power analyses (*t*-tests, difference between two independent means; G*Power software) were performed on FL, SL, and SSN, with 80% indicating statistical power.

A three-way ANOVA [group (training, control)×cycle frequency (1.5, 2, 3 Hz)×length change (1, 3, 5, 7 mm)] with a Holm-Sidak correction for all pairwise comparisons was used to compare all work loop parameters from the passive and active work loops (work of shortening, work of lengthening, and net work output). An effect of group defined a difference between control and trained rat work loops. Where effects of group were detected, two-tailed *t*-tests were employed for comparisons between trained and control rats in specific work loops.

Regression analyses between net work output and SSN, the coefficient *b* from the fitted passive force-length curves, and absolute and specific maximum isometric force were performed to elucidate which training-induced adaptations contributed to improvements in net work output.

Significance was set at *α<*0.05. Effect sizes from three-way ANOVAs are reported as the partial eta squared (η_p_^2^), and from *t*-tests as Cohen's *d* (small effect=0.2, medium effect=0.5, large effect=0.8) where significance was detected*.* Data are reported as mean±s.d. in text and mean±s.e. in figures.

### Ethics statement

All procedures were approved by the Animal Care Committee of the University of Guelph.

## Supplementary Material

Supplementary information

## References

[BIO059491C1] Akagi, R., Hinks, A. and Power, G. A. (2020). Differential changes in muscle architecture and neuromuscular fatigability induced by isometric resistance training at short and long muscle-tendon unit lengths. *J. Appl. Physiol.* 129, 173-184. 10.1152/japplphysiol.00280.2020 32552430 PMC7469237

[BIO059491C2] Alcazar, J., Csapo, R., Ara, I. and Alegre, L. M. (2019). On the shape of the force-velocity relationship in skeletal muscles: the linear, the hyperbolic, and the double-hyperbolic. *Front. Physiol.* 10, 769. 10.3389/fphys.2019.00769 31275173 PMC6593051

[BIO059491C3] Aoki, M. S., Soares, A. G., Miyabara, E. H., Baptista, I. L. and Moriscot, A. S. (2009). Expression of genes related to myostatin signaling during rat skeletal muscle longitudinal growth: myostatin and longitudinal growth. *Muscle Nerve* 40, 992-999. 10.1002/mus.21426 19705480

[BIO059491C4] Baker, J. H. and Hall-Craggs, E. C. B. (1978). Changes in length of sarcomeres following tenotomy of the rat soleus muscle. *Anat. Rec.* 192, 55-58. 10.1002/ar.1091920105 707822

[BIO059491C5] Barclay, C. J. (2005). Modelling diffusive O2 supply to isolated preparations of mammalian skeletal and cardiac muscle. *J. Muscle Res. Cell Motil.* 26, 225-235. 10.1007/s10974-005-9013-x 16322911

[BIO059491C6] Barnett, J. G., Holly, R. G. and Ashmore, C. R. (1980). Stretch-induced growth in chicken wing muscles: biochemical and morphological characterization. *Am. J. Physiol.-Cell Physiol.* 239, C39-C46. 10.1152/ajpcell.1980.239.1.C39 6156603

[BIO059491C7] Baxter, J. R., Hullfish, T. J. and Chao, W. (2018). Functional deficits may be explained by plantarflexor remodeling following Achilles tendon rupture repair: preliminary findings. *J. Biomech.* 79, 238-242. 10.1016/j.jbiomech.2018.08.016 30166224

[BIO059491C8] Binder-Markey, B. I., Sychowski, D. and Lieber, R. L. (2021). Systematic review of skeletal muscle passive mechanics experimental methodology. *J. Biomech.* 129, 110839. 10.1016/j.jbiomech.2021.110839 34736082 PMC8671228

[BIO059491C9] Bogomolovas, J., Fleming, J. R., Franke, B., Manso, B., Simon, B., Gasch, A., Markovic, M., Brunner, T., Knöll, R., Chen, J. et al. (2021). Titin kinase ubiquitination aligns autophagy receptors with mechanical signals in the sarcomere. *EMBO Rep.* 22, e48018. 10.15252/embr.201948018 34402565 PMC8490993

[BIO059491C95] Bonetto, A., Andersson, D. C. and Waning, D. L. (2015). Assessment of muscle mass and strength in mice. BoneKEy Rep. 4, 732. 26331011 10.1038/bonekey.2015.101 PMC4549925

[BIO059491C10] Brashear, S. E., Wohlgemuth, R. P., Gonzalez, G. and Smith, L. R. (2021). Passive stiffness of fibrotic skeletal muscle in mdx mice relates to collagen architecture. *J. Physiol.* 599, 943-962. 10.1113/JP280656 33247944 PMC9926974

[BIO059491C11] Butterfield, T. A. and Herzog, W. (2006). The magnitude of muscle strain does not influence serial sarcomere number adaptations following eccentric exercise. *Pflüg. Arch. Eur. J. Physiol.* 451, 688-700. 10.1007/s00424-005-1503-6 16133258

[BIO059491C12] Butterfield, T. A., Leonard, T. R. and Herzog, W. (2005). Differential serial sarcomere number adaptations in knee extensor muscles of rats is contraction type dependent. *J. Appl. Physiol.* 99, 1352-1358. 10.1152/japplphysiol.00481.2005 15947030

[BIO059491C13] Caiozzo, V. J. and Baldwin, K. M. (1997). Determinants of work produced by skeletal muscle: potential limitations of activation and relaxation. *Am. J. Physiol.-Cell Physiol.* 273, C1049-C1056. 10.1152/ajpcell.1997.273.3.C1049 9316426

[BIO059491C14] Chen, J., Mashouri, P., Fontyn, S., Valvano, M., Elliott-Mohamed, S., Noonan, A. M., Brown, S. H. M. and Power, G. A. (2020). The influence of training-induced sarcomerogenesis on the history dependence of force. *J. Exp. Biol.* 223, jeb218776. 10.1242/jeb.218776 32561632

[BIO059491C96] Cheng, A. J. and Westerblad, H. (2017). Mechanical isolation, and measurement of force and myoplasmic free [Ca2+] in fully intact single skeletal muscle fibers. *Nat. Protoc.* 12, 1763-1776. 28771237 10.1038/nprot.2017.056

[BIO059491C15] Coutinho, E. L., Gomes, A. R. S., França, C. N., Oishi, J. and Salvini, T. F. (2004). Effect of passive stretching on the immobilized soleus muscle fiber morphology. *Braz. J. Med. Biol. Res.* 37, 1853-1861. 10.1590/S0100-879X2004001200011 15558192

[BIO059491C16] Cox, V. M., Williams, P. E., Wright, H., James, R. S., Gillott, K. L., Young, I. S. and Goldspink, D. F. (2000). Growth induced by incremental static stretch in adult rabbit latissimus dorsi muscle. *Exp. Physiol.* 85, 193-202. 10.1111/j.1469-445X.2000.01950.x 10751516

[BIO059491C17] Davis, J. F., Khir, A. W., Barber, L., Reeves, N. D., Khan, T., DeLuca, M. and Mohagheghi, A. A. (2020). The mechanisms of adaptation for muscle fascicle length changes with exercise: implications for spastic muscle. *Med. Hypotheses* 144, 110199. 10.1016/j.mehy.2020.110199 33254508

[BIO059491C18] De Jaeger, D., Joumaa, V. and Herzog, W. (2015). Intermittent stretch training of rabbit plantarflexor muscles increases soleus mass and serial sarcomere number. *J. Appl. Physiol.* 118, 1467-1473. 10.1152/japplphysiol.00515.2014 26078433

[BIO059491C19] Drazan, J. F., Hullfish, T. J. and Baxter, J. R. (2019). Muscle structure governs joint function: linking natural variation in medial gastrocnemius structure with isokinetic plantar flexor function. *Biol. Open* 8, bio048520. 10.1242/bio.048520 31784422 PMC6918776

[BIO059491C20] Farup, J., Kjølhede, T., Sørensen, H., Dalgas, U., Møller, A. B., Vestergaard, P. F., Ringgaard, S., Bojsen-Møller, J. and Vissing, K. (2012). Muscle morphological and strength adaptations to endurance vs. resistance training. *J. Strength Cond. Res.* 26, 398-407. 10.1519/JSC.0b013e318225a26f 22266546

[BIO059491C21] Franchi, M. V., Ruoss, S., Valdivieso, P., Mitchell, K. W., Smith, K., Atherton, P. J., Narici, M. V. and Flück, M. (2018). Regional regulation of focal adhesion kinase after concentric and eccentric loading is related to remodelling of human skeletal muscle. *Acta Physiol.* 223, e13056. 10.1111/apha.13056 29438584

[BIO059491C22] Gans, C. and Bock, W. J. (1965). The functional significance of muscle architecture--a theoretical analysis. *Ergeb Anat Entwicklungsgesch* 38, 115-142. 5319094

[BIO059491C23] Gans, C. and de Vree, F. (1987). Functional bases of fiber length and angulation in muscle. *J. Morphol.* 192, 63-85. 10.1002/jmor.1051920106 3455200

[BIO059491C24] Gillies, A. R. and Lieber, R. L. (2011). Structure and function of the skeletal muscle extracellular matrix: skeletal muscle ECM. *Muscle Nerve* 44, 318-331. 10.1002/mus.22094 21949456 PMC3177172

[BIO059491C25] Gokhin, D. S., Dubuc, E. A., Lian, K. Q., Peters, L. L. and Fowler, V. M. (2014). Alterations in thin filament length during postnatal skeletal muscle development and aging in mice. *Front. Physiol.* 5, 375. 10.3389/fphys.2014.00375 25324783 PMC4178374

[BIO059491C26] Gollapudi, S. K. and Lin, D. C. (2009). Experimental determination of sarcomere force–length relationship in type-I human skeletal muscle fibers. *J. Biomech.* 42, 2011-2016. 10.1016/j.jbiomech.2009.06.013 19647260

[BIO059491C27] Gordon, A. M., Huxley, A. F. and Julian, F. J. (1966a). The variation in isometric tension with sarcomere length in vertebrate muscle fibres. *J. Physiol.* 184, 170-192. 10.1113/jphysiol.1966.sp007909 5921536 PMC1357553

[BIO059491C28] Gordon, A. M., Huxley, A. F. and Julian, F. J. (1966b). Tension development in highly stretched vertebrate muscle fibres. *J. Physiol.* 184, 143-169. 10.1113/jphysiol.1966.sp007908 5921535 PMC1357552

[BIO059491C29] Han, X.-Y., Wang, W., Koskinen, S. O. A., Kovanen, V., Takala, T. E. S., Komulainen, J., Vihko, V. and Trackman, P. C. (1999). Increased mRNAs for procollagens and key regulating enzymes in rat skeletal muscle following downhill running. *Pflüg. Arch. Eur. J. Physiol.* 437, 857-864. 10.1007/s004240050855 10370063

[BIO059491C30] Heinemeier, K. M., Olesen, J. L., Haddad, F., Langberg, H., Kjaer, M., Baldwin, K. M. and Schjerling, P. (2007). Expression of collagen and related growth factors in rat tendon and skeletal muscle in response to specific contraction types: collagen and TGF-β-1 expression in exercised tendon and muscle. *J. Physiol.* 582, 1303-1316. 10.1113/jphysiol.2007.127639 17540706 PMC2075262

[BIO059491C31] Herbert, R. D. and Balnave, R. J. (1993). The effect of position of immobilisation on resting length, resting stiffness, and weight of the soleus muscle of the rabbit. *J. Orthop. Res.* 11, 358-366. 10.1002/jor.1100110307 8326442

[BIO059491C32] Herbert, R. D. and Gandevia, S. C. (2019). The passive mechanical properties of muscle. *J. Appl. Physiol.* 126, 1442-1444. 10.1152/japplphysiol.00966.2018 30412027

[BIO059491C33] Herring, S. W., Grimm, A. F. and Grimm, B. R. (1984). Regulation of sarcomere number in skeletal muscle: a comparison of hypotheses. *Muscle Nerve* 7, 161-173. 10.1002/mus.880070213 6717493

[BIO059491C34] Herzog, W. and Fontana, H. B. (2022). Does eccentric exercise stimulate sarcomerogenesis? *J. Sport Health Sci.* 11, 40-42. 10.1016/j.jshs.2021.10.001 34695612 PMC8847948

[BIO059491C35] Heslinga, J. W. and Huijing, P. A. (1993). Muscle length-force characteristics in relation to muscle architecture: a bilateral study of gastrocnemius medialis muscles of unilaterally immobilized rats. *Eur. J. Appl. Physiol.* 66, 289-298. 10.1007/BF00237771 8495688

[BIO059491C36] Hill, C., James, R., Cox, V. and Tallis, J. (2019). Does dietary-induced obesity in old age impair the contractile performance of isolated mouse soleus, extensor digitorum longus and diaphragm skeletal muscles? *Nutrients* 11, 505. 10.3390/nu11030505 PMC6470722 30818814

[BIO059491C37] Huijing, P. A. (1999). Muscle as a collagen fiber reinforced composite: a review of force transmission in muscle and whole limb. *J. Biomech.* 32, 329-345. 10.1016/S0021-9290(98)00186-9 10213024

[BIO059491C38] Hyldahl, R. D. and Hubal, M. J. (2014). Lengthening our perspective: morphological, cellular, and molecular responses to eccentric exercise: exercise-induced muscle damage. *Muscle Nerve* 49, 155-170. 10.1002/mus.24077 24030935

[BIO059491C39] Jakubiec-Puka, A. and Carraro, U. (1991). Remodelling of the contractile apparatus of striated muscle stimulated electrically in a shortened position. *J. Anat.* 178, 83-100. 1810938 PMC1260537

[BIO059491C40] James, R. S., Altringham, J. D. and Goldspink, D. F. (1995). The mechanical properties of fast and slow skeletal muscles of the mouse in relation to their locomotory function. *J. Exp. Biol.* 198, 491-502. 10.1242/jeb.198.2.491 7699317

[BIO059491C41] James, R. S., Young, I. S., Cox, V. M., Goldspink, D. F. and Altringham, J. D. (1996). Isometric and isotonic muscle properties as determinants of work loop power output. *Pflüg. Arch. Eur. J. Physiol.* 432, 767-774. 10.1007/s004240050197 8772125

[BIO059491C42] Jorgenson, K. W., Phillips, S. M. and Hornberger, T. A. (2020). Identifying the structural adaptations that drive the mechanical load-induced growth of skeletal muscle: a scoping review. *Cells* 9, 1658. 10.3390/cells9071658 PMC7408414 32660165

[BIO059491C43] Josephson, R. K. (1999). Dissecting muscle power output. *J. Exp. Biol.* 202, 3369-3375. 10.1242/jeb.202.23.3369 10562519

[BIO059491C44] Josephson, R. K. and Darrell, S. R. (1989). Strain, muscle length and work output in a crab muscle. *J. Exp. Biol.* 145, 45-61. 10.1242/jeb.145.1.45

[BIO059491C45] Karpakka, J. A., Pesola, M. K. and Takala Timo, E. S. (1992). The effects of anabolic steroids on collagen synthesis in rat skeletal muscle and tendon: a preliminary report. *Am. J. Sports Med.* 20, 262-266. 10.1177/036354659202000305 1636855

[BIO059491C46] Kawakami, Y. and Lieber, R. L. (2000). Interaction between series compliance and sarcomere kinetics determines internal sarcomere shortening during fixed-end contraction. *J. Biomech.* 33, 1249-1255. 10.1016/S0021-9290(00)00095-6 10899334

[BIO059491C47] Kjær, M. (2004). Role of extracellular matrix in adaptation of tendon and skeletal muscle to mechanical loading. *Physiol. Rev.* 84, 649-698. 10.1152/physrev.00031.2003 15044685

[BIO059491C48] Koh, T. J. (1995). Do adaptations in serial sarcomere number occur with strength training? *Hum. Mov. Sci.* 14, 61-77. 10.1016/0167-9457(94)00047-I

[BIO059491C49] Koh, T. J. and Tidball, J. G. (1999). Nitric oxide synthase inhibitors reduce sarcomere addition in rat skeletal muscle. *J. Physiol.* 519, 189-196. 10.1111/j.1469-7793.1999.0189o.x 10432349 PMC2269494

[BIO059491C50] Lichtwark, G. A. and Barclay, C. J. (2010). The influence of tendon compliance on muscle power output and efficiency during cyclic contractions. *J. Exp. Biol.* 213, 707-714. 10.1242/jeb.038026 20154185

[BIO059491C51] Lieber, R. L. and Ward, S. R. (2011). Skeletal muscle design to meet functional demands. *Philos. Trans. R. Soc. B Biol. Sci.* 366, 1466-1476. 10.1098/rstb.2010.0316 PMC3130443 21502118

[BIO059491C52] Lieber, R. L., Yeh, Y. and Baskin, R. J. (1984). Sarcomere length determination using laser diffraction. Effect of beam and fiber diameter. *Biophys. J.* 45, 1007-1016. 10.1016/S0006-3495(84)84246-0 6610443 PMC1434983

[BIO059491C53] Lutz, G. J. and Rome, L. C. (1994). Built for jumping: the design of the frog muscular system. *Science* 263, 370-372. 10.1126/science.8278808 8278808

[BIO059491C54] Lynn, R., Talbot, J. A. and Morgan, D. L. (1998). Differences in rat skeletal muscles after incline and decline running. *J. Appl. Physiol.* 85, 98-104. 10.1152/jappl.1998.85.1.98 9655761

[BIO059491C94] Ma, W. and Irving, T. C. (2019). X-ray Diffraction of Intact Murine Skeletal Muscle as a Tool for Studying the Structural Basis of Muscle Disease. *J. Vis. Exp. JoVE*. 10.3791/59559 PMC6765332 31380854

[BIO059491C55] Macadam, P., Cronin, J. B. and Feser, E. H. (2022). Acute and longitudinal effects of weighted vest training on sprint-running performance: a systematic review. *Sports Biomech.* 21, 239-254. 10.1080/14763141.2019.1607542 31070108

[BIO059491C56] Mashouri, P., Chen, J., Noonan, A. M., Brown, S. H. M. and Power, G. A. (2021). Modifiability of residual force depression in single muscle fibers following uphill and downhill training in rats. *Physiol. Rep.* 9, e14725. 10.14814/phy2.14725 33502825 PMC7839327

[BIO059491C57] Mohammed, G. A. and Hou, M. (2016). Optimization of active muscle force–length models using least squares curve fitting. *IEEE Trans. Biomed. Eng.* 63, 630-635. 10.1109/TBME.2015.2467169 26276984

[BIO059491C58] Moo, E. K., Fortuna, R., Sibole, S. C., Abusara, Z. and Herzog, W. (2016). In vivo sarcomere lengths and sarcomere elongations are not uniform across an intact muscle. *Front. Physiol.* 7, 187. 10.3389/fphys.2016.00187 27252660 PMC4879144

[BIO059491C59] Morais, G. P., da Rocha, A. L., Neave, L. M., de A. Lucas, G., Leonard, T. R., Carvalho, A., da Silva, A. S. R. and Herzog, W. (2020). Chronic uphill and downhill exercise protocols do not lead to sarcomerogenesis in mouse skeletal muscle. *J. Biomech.* 98, 109469. 10.1016/j.jbiomech.2019.109469 31732175

[BIO059491C60] Nicolopoulos-Stournaras, S. and Iles, J. F. (1984). Hindlimb muscle activity during locomotion in the rat (Rattus norvegicus) (Rodentia: Muridae). *J. Zool.* 203, 427-440. 10.1111/j.1469-7998.1984.tb02342.x

[BIO059491C61] Noonan, A. M., Mashouri, P., Chen, J., Power, G. A. and Brown, S. H. M. (2020). Training induced changes to skeletal muscle passive properties are evident in both single fibers and fiber bundles in the rat Hindlimb. *Front. Physiol.* 11, 907. 10.3389/fphys.2020.00907 32903515 PMC7435064

[BIO059491C62] Ochi, E., Nakazato, K. and Ishii, N. (2007). Effects of eccentric exercise on joint stiffness and muscle connectin (Titin) isoform in the rat Hindlimb. *J. Physiol. Sci.* 57, 1-6. 10.2170/physiolsci.RP008806 17081353

[BIO059491C63] Pansarasa, O., Rinaldi, C., Parente, V., Miotti, D., Capodaglio, P. and Bottinelli, R. (2009). Resistance training of long duration modulates force and unloaded shortening velocity of single muscle fibres of young women. *J. Electromyogr. Kinesiol.* 19, e290-e300. 10.1016/j.jelekin.2008.07.007 18801662

[BIO059491C64] Parente, V., D'Antona, G., Adami, R., Miotti, D., Capodaglio, P., De Vito, G. and Bottinelli, R. (2008). Long-term resistance training improves force and unloaded shortening velocity of single muscle fibres of elderly women. *Eur. J. Appl. Physiol.* 104, 885-893. 10.1007/s00421-008-0845-0 18677504

[BIO059491C65] Penman, K. A. (1970). Human striated muscle ultrastructural changes accompanying increased strength without hypertrophy. *Res. Q.* 41, 418-424. 5272917

[BIO059491C66] Ranatunga, K. (2018). Temperature effects on force and actin–myosin interaction in muscle: a look back on some experimental findings. *Int. J. Mol. Sci.* 19, 1538. 10.3390/ijms19051538 PMC5983754 29786656

[BIO059491C67] Rome, L. C. (1994). 6-The mechanical design of the fish muscular system. In *The Mechanics and Physiology of Animal Swimming* (ed. L. Maddock, Q. Bone and J. M. V. Rayner), pp. 75-98. Cambridge University Press. 10.1017/CBO9780511983641.007

[BIO059491C68] Roots, H. and Ranatunga, K. W. (2008). An analysis of the temperature dependence of force, during steady shortening at different velocities, in (mammalian) fast muscle fibres. *J. Muscle Res. Cell Motil.* 29, 9-24. 10.1007/s10974-008-9138-9 18523851 PMC2493522

[BIO059491C69] Roy, R. R., Meadows, I. D., Baldwin, K. M. and Edgerton, V. R. (1982). Functional significance of compensatory overloaded rat fast muscle. *J. Appl. Physiol.* 52, 473-478. 10.1152/jappl.1982.52.2.473 7061301

[BIO059491C70] Roy, R. R., Hutchison, D. L., Pierotti, D. J., Hodgson, J. A. and Edgerton, V. R. (1991). EMG patterns of rat ankle extensors and flexors during treadmill locomotion and swimming. *J. Appl. Physiol.* 70, 2522-2529. 10.1152/jappl.1991.70.6.2522 1885445

[BIO059491C71] Salzano, M. Q., Cox, S. M., Piazza, S. J. and Rubenson, J. (2018). American society of biomechanics journal of biomechanics award 2017: high-acceleration training during growth increases optimal muscle fascicle lengths in an avian bipedal model. *J. Biomech.* 80, 1-7. 10.1016/j.jbiomech.2018.09.001 30266195 PMC6201240

[BIO059491C72] Schaeffer, P. J. and Lindstedt, S. L. (2013). How animals move: comparative lessons on animal locomotion. *Compr. Physiol.* 3, 289-314. 10.1002/cphy.c110059 23720288

[BIO059491C73] Schilder, R. J., Kimball, S. R., Marden, J. H. and Jefferson, L. S. (2011). Body weight-dependent troponin T alternative splicing is evolutionarily conserved from insects to mammals and is partially impaired in skeletal muscle of obese rats. *J. Exp. Biol.* 214, 1523-1532. 10.1242/jeb.051763 21490260 PMC3076076

[BIO059491C74] Shrager, J. B., Kim, D.-K., Hashmi, Y. J., Stedman, H. H., Zhu, J., Kaiser, L. R. and Levine, S. (2002). Sarcomeres are added in series to emphysematous rat diaphragm after lung volume reduction surgery. *Chest* 121, 210-215. 10.1378/chest.121.1.210 11796453

[BIO059491C75] Soares, A. G., Aoki, M. S., Miyabara, E. H., DeLuca, C. V., Ono, H. Y., Gomes, M. D. and Moriscot, A. S. (2007). Ubiquitin-ligase and deubiquitinating gene expression in stretched rat skeletal muscle. *Muscle Nerve* 36, 685-693. 10.1002/mus.20866 17657803

[BIO059491C76] Spletter, M. L., Barz, C., Yeroslaviz, A., Zhang, X., Lemke, S. B., Bonnard, A., Brunner, E., Cardone, G., Basler, K., Habermann, B. H. et al. (2018). A transcriptomics resource reveals a transcriptional transition during ordered sarcomere morphogenesis in flight muscle. *Elife* 7, e34058. 10.7554/eLife.34058 29846170 PMC6005683

[BIO059491C77] Sugama, S., Tachino, K. and Haida, N. (1999). Effect of immobilization on solubility of soleus and gastrocnemius muscle collagen. biochemical studies on collagen from soleus and gastrocnemius muscles of rat. *J. JPN. Phys. Ther. Assoc.* 2, 25-29. 10.1298/jjpta.2.25 25792910 PMC4316489

[BIO059491C78] Swoap, S. J., Caiozzo, V. J. and Baldwin, K. M. (1997). Optimal shortening velocities for in situ power production of rat soleus and plantaris muscles. *Am. J. Physiol.-Cell Physiol.* 273, C1057-C1063. 10.1152/ajpcell.1997.273.3.C1057 9316427

[BIO059491C79] Tabary, J. C., Tabary, C., Tardieu, C., Tardieu, G. and Goldspink, G. (1972). Physiological and structural changes in the cat's soleus muscle due to immobilization at different lengths by plaster casts*. *J. Physiol.* 224, 231-244. 10.1113/jphysiol.1972.sp009891 5039983 PMC1331536

[BIO059491C80] Takahashi, M., Ward, S. R., Fridén, J. and Lieber, R. L. (2012). Muscle excursion does not correlate with increased serial sarcomere number after muscle adaptation to stretched tendon transfer: functional adaptation after tendon transfer. *J. Orthop. Res.* 30, 1774-1780. 10.1002/jor.22137 22532301 PMC3407307

[BIO059491C81] Turrina, A., Martínez-González, M. A. and Stecco, C. (2013). The muscular force transmission system: role of the intramuscular connective tissue. *J. Bodyw. Mov. Ther.* 17, 95-102. 10.1016/j.jbmt.2012.06.001 23294690

[BIO059491C82] Walker, S. M. and Schrodt, G. R. (1974). I segment lengths and thin filament periods in skeletal muscle fibers of the rhesus monkey and the human. *Anat. Rec.* 178, 63-81. 10.1002/ar.1091780107 4202806

[BIO059491C83] Ward, S. R. and Lieber, R. L. (2005). Density and hydration of fresh and fixed human skeletal muscle. *J. Biomech.* 38, 2317-2320. 10.1016/j.jbiomech.2004.10.001 16154420

[BIO059491C84] Wickiewicz, T. L., Roy, R. R., Powell, P. L., Perrine, J. J. and Edgerton, V. R. (1984). Muscle architecture and force-velocity relationships in humans. *J. Appl. Physiol.* 57, 435-443. 10.1152/jappl.1984.57.2.435 6469814

[BIO059491C85] Williams, P. E. (1990). Use of intermittent stretch in the prevention of serial sarcomere loss in immobilised muscle. *Ann. Rheum. Dis.* 49, 316-317. 10.1136/ard.49.5.316 2344211 PMC1004076

[BIO059491C86] Williams, P. E. and Goldspink, G. (1973). The effect of immobilization on the longitudinal growth of striated muscle fibres. *J. Anat.* 116, 45-55. 4798240 PMC1271549

[BIO059491C87] Williams, P. E. and Goldspink, G. (1978). Changes in sarcomere length and physiological properties in immobilized muscle. *J. Anat.* 127, 459-468. 744744 PMC1235732

[BIO059491C88] Woittiez, R. D., Baan, G. C., Huijing, P. A. and Rozendal, R. H. (1985). Functional characteristics of the calf muscles of the rat. *J. Morphol.* 184, 375-387. 10.1002/jmor.1051840311 4057263

[BIO059491C89] Wong, T. S. and Booth, F. W. (1988). Skeletal muscle enlargement with weight-lifting exercise by rats. *J. Appl. Physiol.* 65, 950-954. 10.1152/jappl.1988.65.2.950 2459101

[BIO059491C90] Zajac, F. E. (1989). Muscle and tendon: properties, models, scaling, and application to biomechanics and motor control. *Crit. Rev. Biomed. Eng.* 17, 359-411. 2676342

[BIO059491C91] Zhu, W. G., Hibbert, J. E., Lin, K.-H., Steinert, N. D., Lemens, J. L., Jorgenson, K. W., Newman, S. M., Lamming, D. W. and Hornberger, T. A. (2021). Weight pulling: a novel mouse model of human progressive resistance exercise. *Cells* 10, 2549. 10.3390/cells10092459 34572107 PMC8465477

[BIO059491C92] Zimmerman, S. D., McCormick, R. J., Vadlamudi, R. K. and Thomas, D. P. (1993). Age and training alter collagen characteristics in fast- and slow-twitch rat limb muscle. *J. Appl. Physiol.* 75, 1670-1674. 10.1152/jappl.1993.75.4.1670 8282619

[BIO059491C93] Zöllner, A. M., Abilez, O. J., Böl, M. and Kuhl, E. (2012). Stretching skeletal muscle: chronic muscle lengthening through sarcomerogenesis. *PLoS One* 7, e45661. 10.1371/journal.pone.0045661 23049683 PMC3462200

