# Supplementary material for: Influence of weighted downhill running training on serial sarcomere number and work loop performance in the rat soleus
Source: Biol Open. 2022 Jul 25;11(7):bio059491. doi: 10.1242/bio.059491 (PMC9346294; doi:10.1242/bio.059491)
Supplement: Supplementary information [file biolopen-11-059491-s1.pdf]

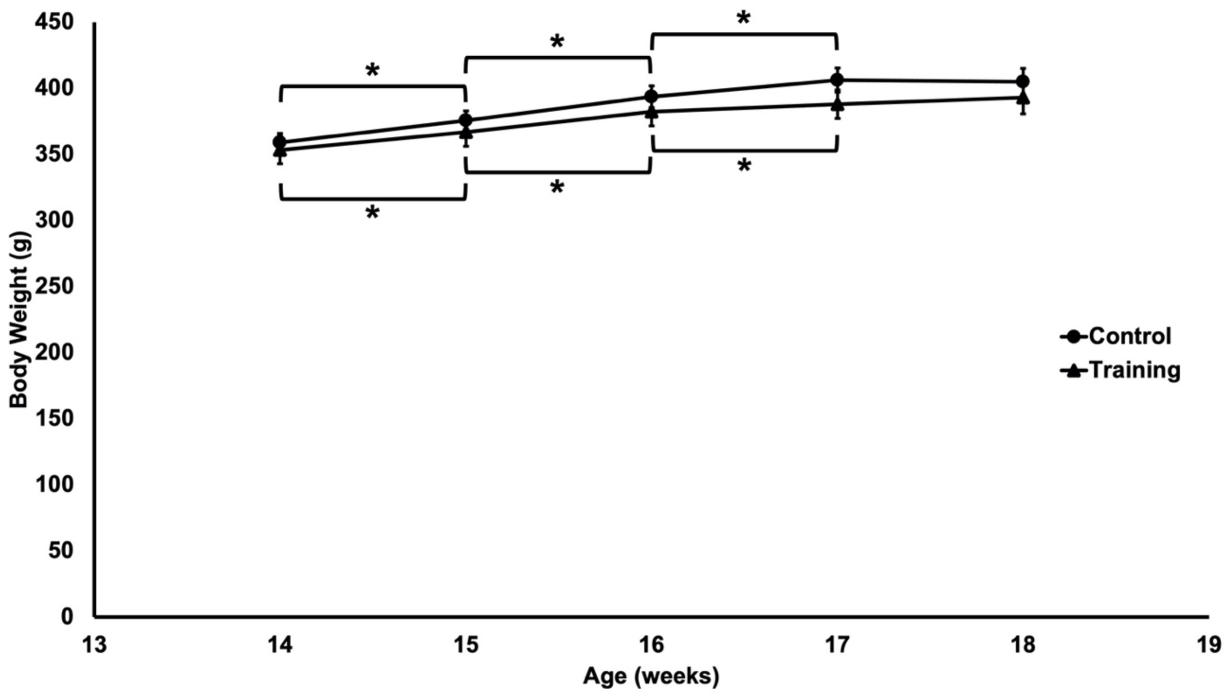

Fig. S1. Changes in rat body weight from the start of the training period (age ~14 weeks) to the day of sacrifice (age ~18 weeks) (n = 18 control, n = 14 training). Data are reported as mean ± standard error. *Significant difference between time points.

Figure S1 shows body weight from the start of the training period to sacrifice. In control and trained rats, body weight increased up to 17 weeks of age (week 4 of training) (all comparisons $P < 0.01$), then plateaued from 17 to 18 weeks of age (control: $P = 0.74$, trained: $P = 0.09$). While visually it appears that trained rats tended to weigh increasingly less than controls across the training period, there were no differences in body weight between trained and control rats at any weeks (P = 0.20-0.50). This observation strengthens comparability between the training and control groups, as it discounts differences in body weight as a confounding variable.

Biology Open • Supplementary information

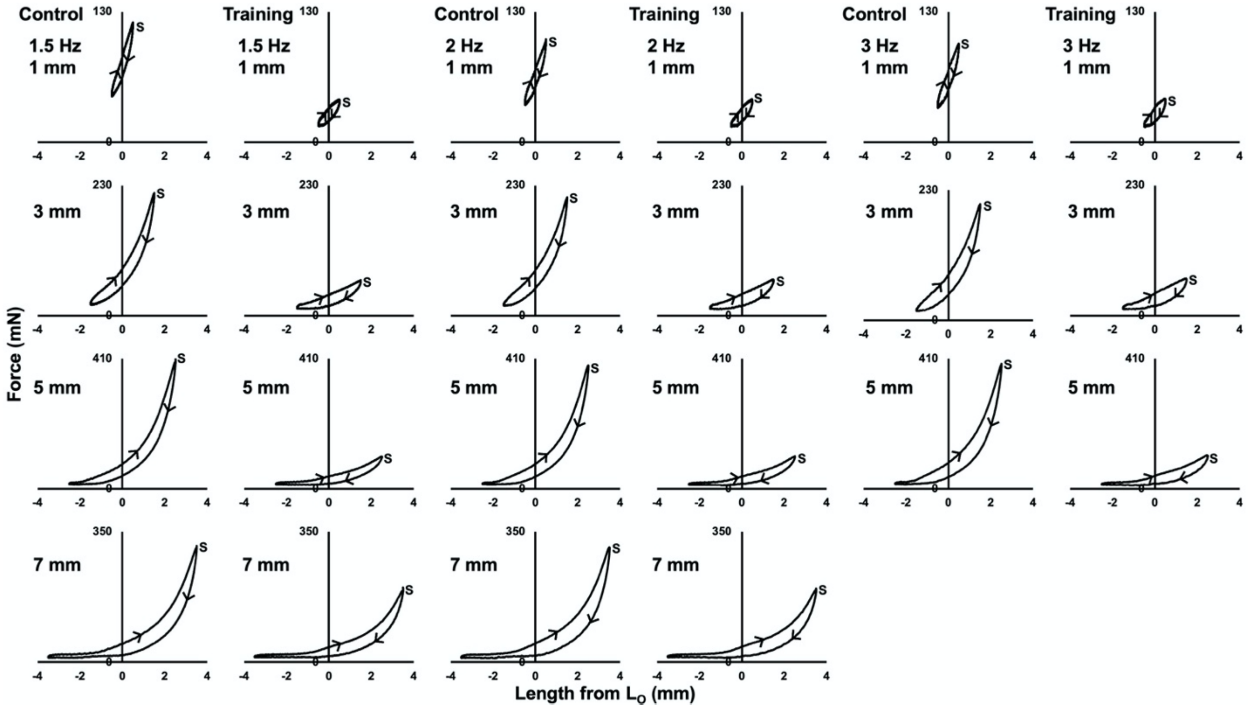

Fig. S2. Representative passive (i.e., no stimulation) work loop traces for 1 control and 1 trained rat across cycle frequencies of 1.5, 2, and 3 Hz, and length changes of 1, 3, 5, and 7 mm. *S* indicates the start of the cycle. Arrows indicate the direction of the cycle.

A: If trained rats had the same SL at L_O as controls (i.e., 2.81 µm) and experienced the same observed increase in SSN

Hypothetical trained FL = trained SSN / control SL

$= 6024$ sarcomeres / (2.81×10^{-3} mm)

= 16.927 mm

	Control (measured resting length at L_O: 2.81 µm)			Trained (if measured resting length at L_O was 2.81 µm)		
**Length change**	SL range	SL excursion (% of SL at L_O)	Shortening velocity at 1.5 Hz	SL range	Excursion from SL at L_O	Shortening velocity at 1.5 Hz
1 mm	2.71-2.83 µm	4.27%	0.13 SL/s	2.76-2.86 µm	3.56%	0.11 SL/s
3 mm	2.60-2.95 µm	12.46%	0.37 SL/s	2.65-2.97 µm	11.39%	0.34 SL/s

L_O: optimal muscle length; SL: sarcomere length

B: If trained rats had the same SL at L_O as controls (i.e., 2.81 µm) to achieve the same observed increase in FL

Hypothetical trained SSN = trained FL / control SL

= 17.523 mm / (2.81×10^{-3} mm)

= 6236 sarcomeres

	Control (measured resting length at L_O: 2.81 µm)			Trained (if measured resting length at L_O was 2.81 µm)		
**Length change**	SL range	SL excursion (% of SL at L_O)	Shortening velocity at 1.5 Hz	SL range	Excursion from SL at L_O	Shortening velocity at 1.5 Hz
1 mm	2.71-2.83 µm	4.27%	0.13 SL/s	2.76-2.86 µm	3.56%	0.11 SL/s
3 mm	2.60-2.95 µm	12.46%	0.37 SL/s	2.65-2.97 µm	11.39%	0.34 SL/s

C: If trained rats experienced a much greater (33%) increase in SSN and had the same SL at L_O as controls

Trained SSN as 33% greater than control SSN = control SSN × 1.33

$= 5577 \times 1.33$

= 7417 sarcomeres

Hypothetical trained FL = trained SSN × control SL

$= 7417 \times (2.81 \times 10^{-3}\ \text{mm})$

= 20.842 mm

	**Control (measured resting length at L_O: 2.81 µm)**			**Trained (if measured resting length at L_O was 2.81 µm)**		
**Length change**	SL range	SL excursion (% of SL at L_O)	Shortening velocity at 1.5 Hz	SL range	Excursion from SL at L_O	Shortening velocity at 1.5 Hz
1 mm	2.71-2.83 µm	4.27%	0.13 SL/s	2.77-2.85 µm	2.85%	0.09 SL/s
3 mm	2.60-2.95 µm	12.46%	0.37 SL/s	2.81-2.94 µm	4.63%	0.14 SL/s

Fig. S3. Estimations for sarcomere excursion relative to sarcomere length (SL) at optimal muscle length (L_O) if trained rats had the same SL as controls (A) to achieve the same observed increase in serial sarcomere number (SSN), (B) to achieve the same observed increase in fascicle length (FL), and (C) if they had experienced a much greater increase in SSN.

Biology Open • Supplementary information
